# Is patient insurance type related to physician recommendation, administration and referral for adult vaccination? A survey of US physicians

**DOI:** 10.1080/21645515.2019.1582402

**Published:** 2019-03-20

**Authors:** Michelle McNamara, Philip O. Buck, Songkai Yan, Leonard R. Friedland, Kristin Lerch, Alysa Murphy, Cosmina Hogea

**Affiliations:** a Adelphi Research, Doylestown, PA, USA; b US Health Outcomes & Epidemiology, Vaccines, GSK, Philadelphia, PA, USA; c US Medical Affairs, Vaccines, GSK, Philadelphia, PA, USA

**Keywords:** Adult vaccines, vaccine recommendations, physician preferences, influenza, Tdap, zoster

## Abstract

This study evaluated physician practices and perceived barriers for influenza, tetanus, diphtheria, pertussis (Tdap), and zoster vaccination of adults in the United States (US), with emphasis on patients with Medicare versus commercial insurance. A cross-sectional internet-based survey of board-certified general/family practitioners and internists (N = 1,000) recruited from a national US physician panel was conducted in May 2017. For influenza, rates of physician recommendation (84% of Medicare patients, 82% of commercially-insured patients), administration (80% Medicare, 78% commercial), and referral (11% Medicare, 11% commercial) were similar regardless of insurance type. Tdap recommendation was higher for commercial compared to Medicare patients (59% vs. 54%, p < 0.001); while zoster recommendation was higher for Medicare patients than commercial (59% vs. 55%, p < 0.001). For Tdap and zoster, higher administration rates were reported in commercial patients (64% Tdap, 36% zoster) than Medicare (56% Tdap, 32% zoster), and referral rates were higher for Medicare patients (19% Tdap, 49% zoster) than commercial (14% Tdap, 42% zoster). Over 40% of physicians would be much more likely to administer Tdap and zoster vaccines if they were covered under Medicare Part B, with more physicians indicating financial barriers as “major” or “moderate” for Medicare than commercial patients. These differences may be related to financial barriers associated with adult vaccinations that are covered under Medicare Part D and involve patient out-of-pocket costs. Efforts to reduce financial barriers associated with adult vaccinations covered under Medicare Part D and to improve patient and physician knowledge could positively impact physician recommendation, administration, and referral for adult vaccination in the US.

## Introduction

In the United States (US), an estimated 14.1 million cases of vaccine-preventable diseases were attributable to unvaccinated adults aged 19 years and older in 2015. Direct costs and productivity losses from vaccine-preventable diseases amounted to a total financial burden of US Dollar (USD) 9 billion, of which approximately 80% was due to unvaccinated adults.^1^ Per Advisory Committee on Immunization Practices (ACIP) recommendations, all adults aged 19 years or older should be given one dose of the influenza vaccine annually. In addition, one dose of tetanus toxoid, reduced diphtheria toxoid, and acellular pertussis vaccine (Tdap) is recommended for adults who did not receive a dose of Tdap as an adult or child and should be followed by one dose of tetanus and diphtheria toxoids (Td) booster every 10 years. Also, ACIP recommendation for herpes zoster vaccination of all adults 60 years and above has been extended to adults aged 50–59 years, effective February 2018.^2^

Despite the routine recommendations, adult immunization coverage remains low. Published estimates based on the National Health Interview Survey (NHIS) 2016 data indicate that 43.5% and 26.6% of adults over the age of 19 years were vaccinated against influenza and Tdap, respectively, whereas 33.4% of adults over the age of 60 years were vaccinated against herpes zoster.^3^ The low adult vaccination coverage rates have been attributed to lack of knowledge and awareness about immunization in patients and health care professionals (HCPs), lack of accessibility, vaccination costs, lack of an efficient and accessible vaccination tracking and reminder systems, HCPs reluctance to store vaccines, vaccine shortages, lack of recommendations by a HCP and lack of insurance coverage.^4,5^ Knowledge, attitudes and practices of physicians play a critical role in vaccine uptake as shown in studies conducted between 1982 and 2010.^6-19^ Health insurance status and coverage further contribute to disparities in adult vaccination.^20^ This is of particular interest given the recent policy changes in the US (e.g. the Affordable Care Act and changes to Medicare).^21,22^ Medicare, a federal health insurance system administered by the Centers for Medicare & Medicaid Services (CMS), covers US citizens who are 65 years and older, and below 65 years who are suffering from a certain disability. Medicare has four parts, ranging from A to D, of which Part B covers the full cost of some vaccines (including vaccines against influenza).^23^ Medicare part B also covers the administration fees for the covered vaccines.^23^ On the other hand, patients enrolled under Medicare Part D will have access to most commercially-available vaccinations not covered under Part B, such as Tdap and zoster,^22,23^ but there may be a copayment to receive these vaccines. The copay amount varies based on where a patient gets vaccinated and the type and phase of insurance (deductible, copayment or coinsurance or catastrophic coverage).^22,24^ Copayment has been identified as a possible hurdle to vaccine uptake among the elderly.^13^ In 2011, CMS incorporated a new tier in Medicare Part D (Tier 6), which would allow the provision of some vaccines without copay. However, few health plans currently employ Tier 6 for their members.^25^

The differences between Medicare and commercial insurance may lead to differences in physician recommendation, referral, and administration of adult vaccines. To our knowledge, no studies have examined this relationship. In this study, we aimed to gain insights on physician knowledge, practices, and perceived barriers for influenza, Tdap and zoster adult vaccination and assessed the differences in vaccine recommendation, administration and referral by physicians based on patient insurance coverage type (commercial or Medicare).

## Results

A convenience sample of 1,000 physicians completed the survey: 500 general practice (GP)/family practice (FP) physicians and 500 internal medicine (IM) physicians, relatively evenly distributed across the US with a mean age of 49.2 years and an average of 17.1 years practicing medicine since residency. Nearly all (99.4%) indicated they were board-certified in their primary specialty. Approximately 70% of physicians spent most of their time treating patients in private practice. Over half of physicians practiced in the suburbs and had large group practices. Furthermore, almost half of the physicians had practices that were independent or were part of integrated health systems that included hospitals, practices and other healthcare settings (Table 1).

**Table 1.** Physician characteristics (N = 1,000).

Physician characteristics	Summary statistics, n (%)
**Specialty (primary profession)**	
Internist	500 (50.0)
Family Practitioner	444 (44.4)
General Practitioner	56 (5.6)
**Board certification**	
Board certified	994 (99.4)
Board eligible	3 (0.3)
Neither certified nor eligible	3 (0.3)
**Gender**	
Male	719 (71.9)
Female	281 (28.1)
**Geographic location**	
Northeast	212 (21.2)
Midwest	231 (23.1)
South	346 (34.6)
West	211 (21.1)
**Physician time practicing since residency, years**	
Mean	17.1
**Physician age, years**	
Mean	49.2
**Setting in which majority of physician time is spent treating patients**	
Private practice	692 (69.2)
Outpatient clinic	211 (21.1)
Hospital (university or academic)	40 (4.0)
Community teaching	38 (3.8)
Community non-teaching	19 (1.9)
**Primary practice location**	
Urban	275 (27.5)
Suburban	588 (58.8)
Rural	137 (13.7)
**Practice type**	
Large group practice	524 (52.4)
Small group practice	306 (30.6)
Solo practice	170 (17.0)
**Practice description**	
Independent	485 (48.5)
Part of an integrated health system that includes hospitals, practices and other healthcare settings	428 (42.8)
Part of an integrated health system that includes other practices like yours only; but not inclusive of hospitals or others	79 (7.9)
Other	8 (0.8)

**Figure 1.**
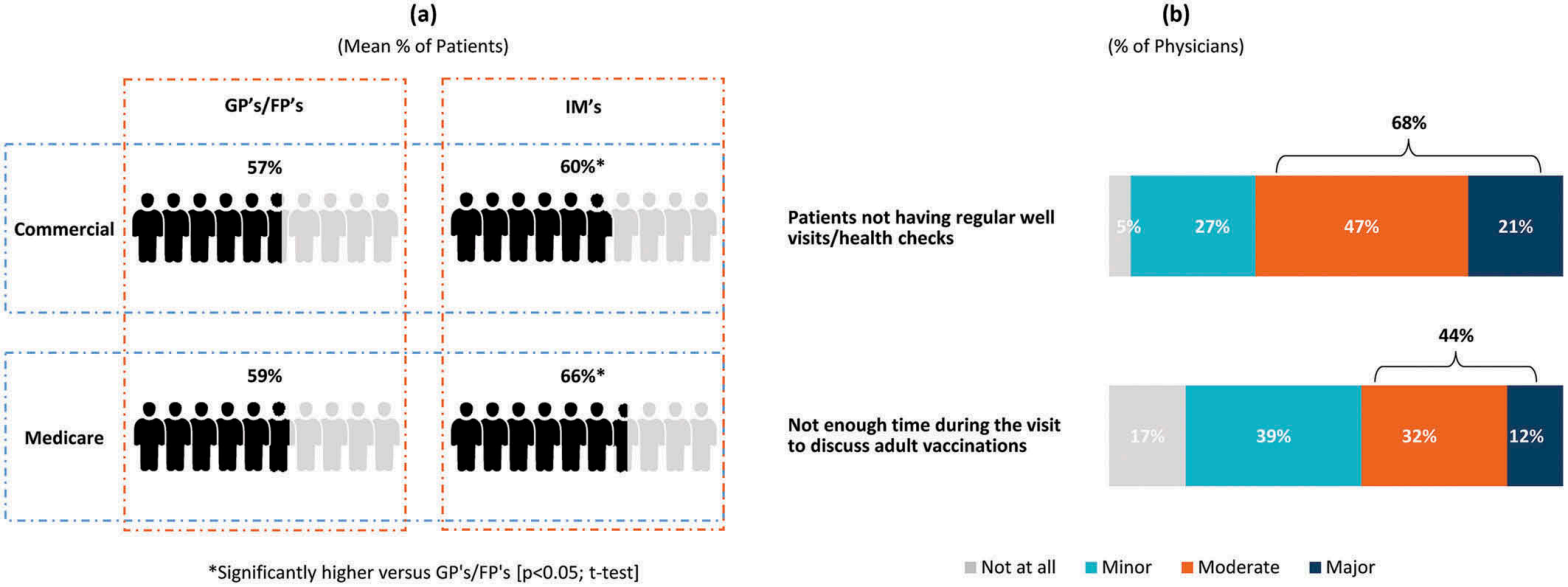
(a) Annual physical/well visits by adult patients, (b) Barriers to adult patients receiving vaccinations. FPs: family practice physicians; GPs: general practitioners; IMs: internal medicine physicians; Total number of Physicians = 1,000

Physicians’ level of involvement related to adult immunization policies and practices varied considerably. Over 85% of physicians were involved to some degree in setting office-wide immunization practices/protocols, determining office vaccination schedules and selecting vaccines, with about half (44.8–48.2%) being very or fully involved. On average, physicians estimated that of all the adult patients they saw at their practice in a typical year, 55.8% were covered by commercial insurance and 52.3% were covered by Medicare (not mutually exclusive) (please refer to Supplement Table 1 for physician-reported patient characteristics). About 6 in 10 adult patients reportedly had a regular (“annual/periodic”) physical/well visit (Figure 1(a)). Regardless of insurance type, GP/FPs reported fewer patients having annual/periodic physical/well visits (57.4% for patients with commercial insurance and 58.8% for patients with Medicare) than IM physicians (60.1% for patients with commercial insurance and 65.6% of patients with Medicare); p < 0.05 for each t-test. The absence of regular visits was considered by 68% of physicians a major/moderate barrier to vaccination, while 44% indicated the lack of adequate time to discuss adult vaccinations during a well visit as a “moderate” to “major” barrier (Figure 1(b)). GP/FPs (72%) were more likely than IMs (64%) to evaluate as a major/moderate barrier to vaccination the absence of regular well visit/health checks (p < 0.05; chi-square test).

### Physician attitudes toward and knowledge related to, adult vaccination

#### Physician attitudes

**Figure 2.**
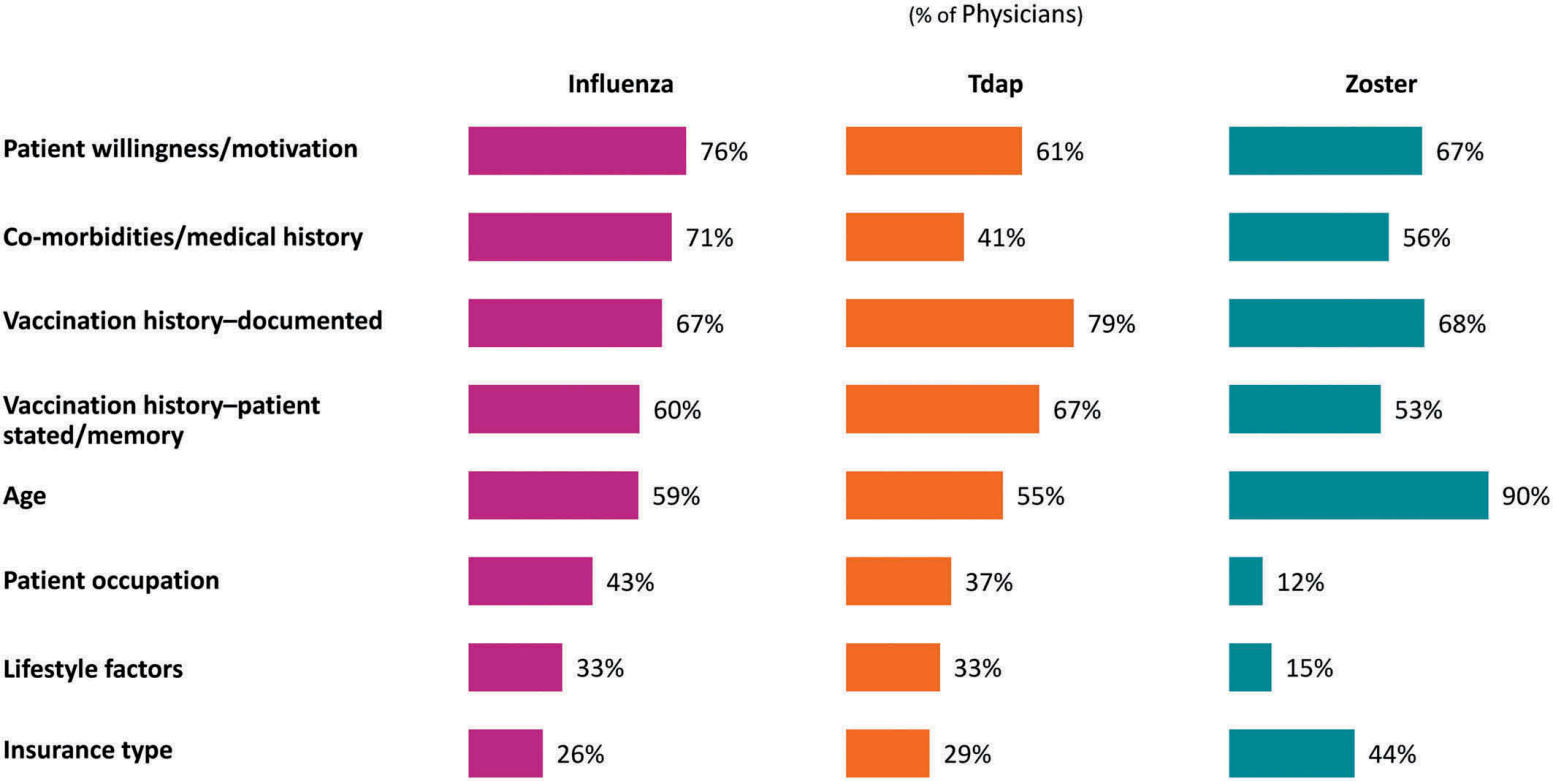
Criteria physicians use to determine patient eligibility for various vaccines. Note: ‘Other’ criteria not shown (Influenza 0.7%; Tdap 0.9%; Zoster 0.4%); Total number of Physicians = 1,000; Tdap, tetanus/diphtheria/acellular pertussis vaccine

The majority of physicians (76.0%) “strongly agree” or “agree” that too many adults in the US suffer from diseases that could be prevented by vaccination, and 74.0% “strongly agree” or “agree” that adult vaccines are not used as much as they should be used (Supplement Figure 1). Most (91.0%) consider it “extremely” or “very” important that all eligible adult patients receive an influenza vaccine; notably fewer consider it “extremely” or “very” important that all eligible adult patients receive a zoster (66.0%) vaccine (data not shown for Tdap due to error in item wording). Physicians most commonly consider patient motivation (influenza: 76.0%; Tdap: 61.0% and zoster: 67.0%) and documented vaccination history (influenza: 67.0%; Tdap: 79.0% and zoster: 68.0%) in assessing their adult patients’ eligibility across all three vaccines (Figure 2).

#### Physician knowledge

The majority of physicians reported being knowledgeable of the ACIP recommendations for adult vaccinations (76.0% for influenza, 67.0% for Tdap, and 67.0% for zoster) (Supplement Figure 2). Routine recommenders more frequently reported being knowledgeable about the recommended ACIP criteria and schedule of administration for adult vaccinations, as compared to non-recommenders (p < 0.05; chi-square test; influenza: 76.4% versus 66.0%; Tdap: 70.6% versus 51.5%; zoster: 70.8% versus 55.7%).

Slightly more than half of physicians (56.0%) correctly identified that influenza vaccine is covered under Medicare Part B, while one-quarter (25.0%) indicated that they “don’t know” the current Medicare coverage for the vaccine. Only about one-quarter (27.0%) correctly identified that Tdap vaccine is covered under Part D and less than half (44.0%) correctly identified that zoster vaccine is covered under Part D. Regarding patients with commercial insurance, 25.0%, 33.0% and 34.0% of physicians indicated that they “don’t know” whether a co-pay is required for influenza, Tdap and zoster vaccinations, respectively (Supplement Figure 3).

**Figure 3.**
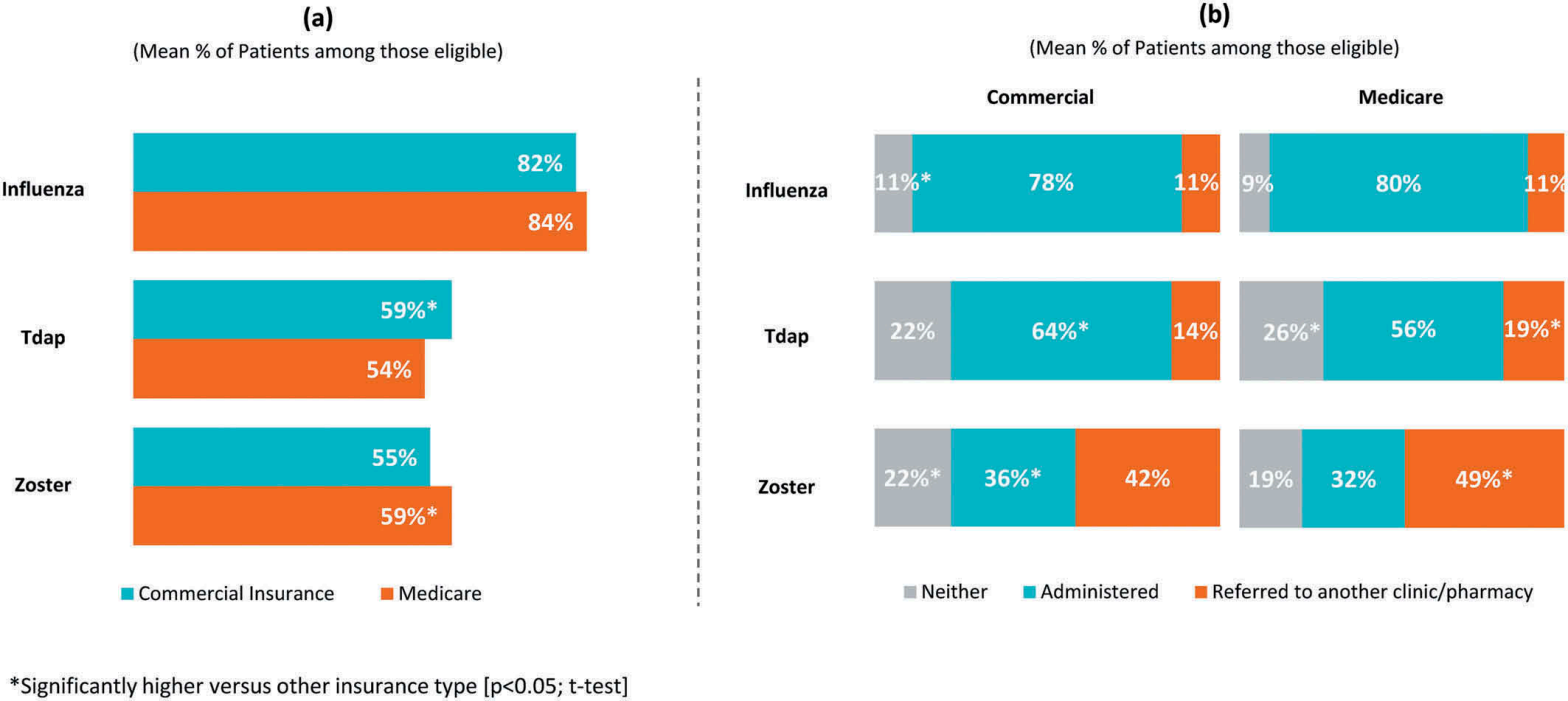
(a) Eligible patients recommended vaccine by insurance type, (b) Eligible patients administered/referred to vaccine by insurance type. Tdap, tetanus/diphtheria/acellular pertussis vaccine; Total number of Physicians = 1,000

### Physician practice with adult vaccinations

During annual physical and well visits with their eligible adult patients, most physicians discuss possible problems from getting sick with the disease if not vaccinated (influenza: 98.0%; zoster: 90.0%); however, fewer discuss or recommend vaccinations (range across vaccines: 60.0%–88.0%) (Supplement Figure 4). A large proportion of physicians (80.0%) “strongly agree” or “agree” that their patients prefer to receive adult vaccinations in the physician’s practice rather than being referred elsewhere for it. Fewer routinely stock Tdap (88.0%) or zoster (52.0%) vaccine in their practice compared to influenza vaccines (98.0%). Only 30.0% “strongly agree” or “agree” that the level of reimbursement they receive for adult vaccinations makes it financially worthwhile to administer them (Supplement Figure 5).

**Figure 4.**
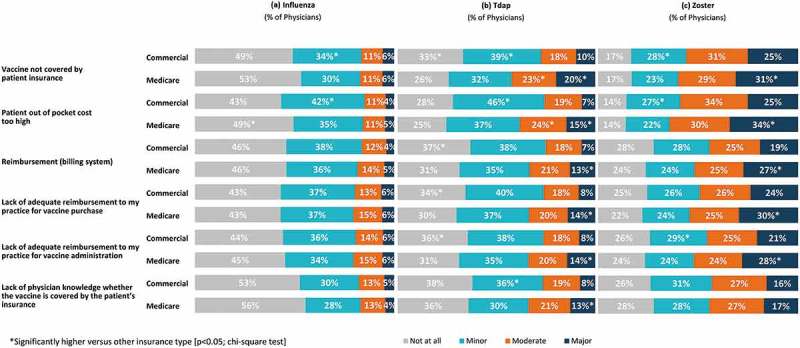
Barriers as identified by physicians to (a) Influenza, (b) Tdap and (c) Zoster vaccinations. Tdap, tetanus/diphtheria/acellular pertussis vaccine; Total number of Physicians = 1,000. Note: Sums of percentages may not equal 100% due to rounding.

**Figure 5.**
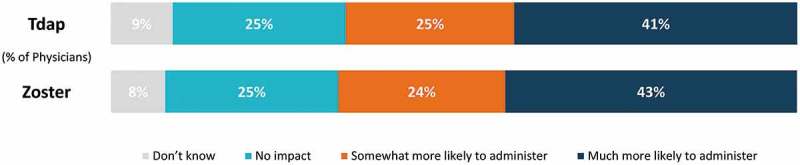
Willingness to administer Tdap and zoster vaccines if covered under Medicare Part B instead of Medicare Part D. Tdap, tetanus/diphtheria/acellular pertussis vaccine; Total number of Physicians = 1,000

Physicians identified patients’ lack of perceived need, concern about adverse events, concern about lack of efficacy, and lack of awareness/knowledge about vaccines and the illness they prevent, as well as the up-front cost of purchasing vaccines and lack of effective reminder system in place for patients and HCPs, as barriers to their eligible adult patients receiving one or more recommended vaccination (Supplement Figure 6).

**Figure 6.**
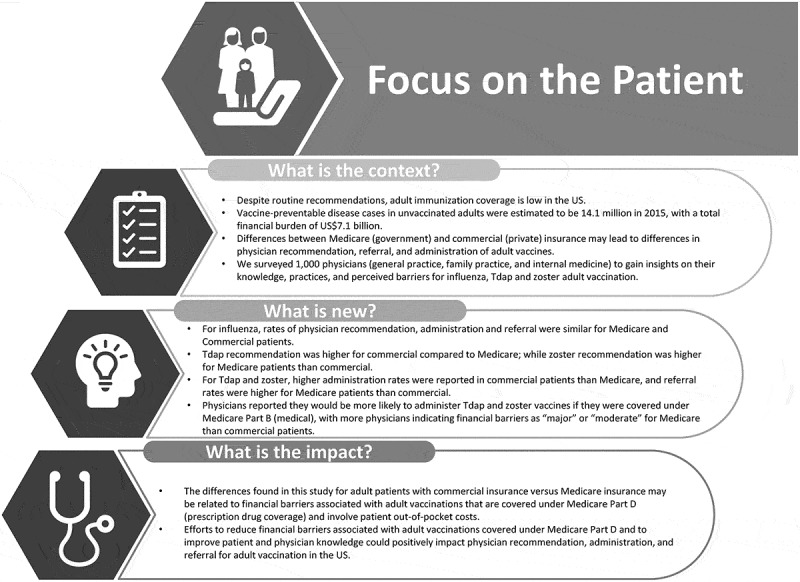
Focus on the patient.

Most physicians (90%) reported using electronic health or medical records (EHR/EMR) to document vaccine administration; among them, while approximately 75% indicated use of EHR/EMR alerts to assess vaccination status for their adult patients, only 25% were required to confirm that they have reviewed their patient’s vaccination status when using the system. Only a very small percentage (10%) used Immunization Information Systems (IIS).

### Rates of recommendation, administration and referral for vaccination, by insurance type

Analysis of the surveyed physicians’ responses regarding the recommendation, administration, and referral rates for adult vaccination indicated that there were some statistically significant differences across the three vaccine types between adult patients with Medicare or commercial insurances.

For influenza, rates of physician recommendation (83.5% of Medicare patients, 81.8% of commercial patients), administration (79.6% of Medicare patients, 78.1% of commercial patients), and referral (11.1% of Medicare patients, 11.0% of commercial patients) were similar regardless of insurance type. While significantly more patients with commercial insurance were recommended Tdap compared to Medicare (59.4% versus 54.4% respectively), recommendation for zoster was significantly higher for patients covered by Medicare (59.4% versus 54.7%). Significantly more patients with commercial insurance compared to Medicare were administered Tdap (64.3% versus 55.6%) and zoster (36.0% versus 32.0%) vaccines and significantly more patients covered by Medicare compared to commercial insurance were referred elsewhere for Tdap (18.6% versus 14.0%) and zoster (49.0% versus 42.0%) (Figure 3; p < 0.05; t-test).

Insurance coverage and financial factors were more frequently identified as moderate/major barriers to vaccinations for patients with Medicare than for patients with commercial insurance, particularly for the Tdap and zoster vaccines (Figure 4; p < 0.05; chi-square test).

GP/FP relied upon their nurses or office staff to make recommendations and/or remind patients about adult vaccination slightly more frequently than did IM physicians (34.6% versus 28.8% (p < 0.05; chi-square test). Significantly more physicians who reported routinely recommending vaccination to their eligible adult patients indicated that they consider it “extremely” or “very” important that all eligible adult patients receive vaccinations, as compared to non-recommenders (routine recommenders versus non-recommenders; p < 0.05; chi-square test); influenza: 92.0% versus 80.0%; zoster: 68.6% versus 56.1%; data not shown for Tdap due to error in item wording).

Overall, physicians stated they would be more likely to administer Tdap and zoster vaccines if covered under Medicare Part B, rather than their current coverage under Part D (Figure 5). Significantly more GP/FPs vs. IMs would be much more likely to administer zoster (46% vs. 39%) and Tdap (45% vs. 38%) (p < 0.05; chi-square test). Compared to non-recommenders, significantly more physicians who reported routinely recommending vaccinations to their eligible adult patients indicated that they would be much more likely to administer the Tdap (43.4% versus 30.1%; p < 0.05) and zoster (43.5% versus 34.1%; p < 0.05) vaccinations in their practice if they were covered under Medicare Part B.

## Discussion

Findings from this survey provide contemporary context and evidence on the importance of understanding current physician attitudes, knowledge, practices and perceived barriers to adult vaccinations in the US. The results are consistent with prior research indicating that physician knowledge, attitudes and preferences influence their decisions about whether to offer or recommend vaccinations to adult patients and that cost, lack of tracking systems, and competing demands are barriers that affect access to immunizations among adults^4,6-12,14-19,26^. Although a recent study from 2016 has explored physician attitudes regarding the perceived importance of vaccines and physician knowledge of ACIP recommendations,^26^ to our knowledge studies have yet to explore physician practices in the context of Medicare versus commercial insurance coverage. The main goal of the present paper was to highlight physicians’ perception and use of adult vaccinations in practice, with focus on examining whether there are differences between patients with Medicare coverage versus patients with commercial insurance coverage. Our survey revealed differences in physician knowledge, practices (recommendation, administration, and referral), and perceived barriers for vaccines by insurance type, as well as gaps identified in physicians’ knowledge relating to patient eligibility for some vaccinations, vaccine insurance coverage and co-payments.

For influenza, physician-reported rates of recommendation, administration, and referral were similar regardless of insurance type. For Tdap, recommendation rates were higher for patients with commercial insurance than for patients with Medicare; while for zoster, recommendation rates were higher for patients with Medicare than for those with commercial insurance. However, that may change in the future given the recent expanded ACIP recommendation for zoster vaccination to include adults starting at the age of 50 years^2^.

For both Tdap and zoster, physician-reported administration rates were higher for patients with commercial insurance than for those with Medicare, and referral rates were higher for patients with Medicare than commercial insurance, despite physician’s belief that patients prefer vaccinations at the doctor’s office. A substantial percentage of physicians indicated that they would be more likely to administer Tdap and zoster if they were covered under Medicare Part B. Financial burden on the provider’s practice (due to high costs of acquisition and storing of the vaccines), as well as failure to assess vaccination status during visits have been recognized as obstacles to the implementation of adult vaccination in several national surveys assessing vaccination practices among general internists and family medicine physicians. Co-pay/coinsurance is recognized as a significant hurdle to the uptake of Tdap and zoster vaccines in Medicare Part D beneficiaries.^13,22,26,27^ Alleviating this financial burden on providers and patients may help improve vaccination rates and consequently reduce morbidity and mortality related to these vaccine-preventable diseases in adults. Policy changes that could help reduce the patient out-of-pocket cost and improve practice reimbursement for vaccine purchase and administration may reduce some of the barriers to adult vaccination, particularly for the more expensive vaccines with Medicare patients.

The opportunity to vaccinate adults may be significantly impacted by lack of patient adherence to annual physical/well visits, as well as insufficient time to address vaccination during the visit. Notably, influenza was much more frequently discussed compared to Tdap and zoster vaccination during office visits. While most physicians agreed that too many adults in the US suffer from diseases that could be prevented by vaccination, the majority also agreed that adult vaccines are not used as much as they should be.

Patient willingness/motivation and vaccination history were most commonly used in practice when assessing adult patients’ eligibility for influenza, Tdap and zoster vaccination. While the majority of physicians reported being knowledgeable of the ACIP recommendations for these three vaccines, considerably fewer were knowledgeable about corresponding insurance coverage.

Fewer physicians routinely stock the zoster vaccine in their practice compared to the Tdap or influenza vaccines, and only approximately one-third of physicians agree that the level of reimbursement they receive for adult vaccinations makes it financially worthwhile for the physicians to administer them. This limitation in stocking the zoster vaccine may be related physicians’ facing challenges in reimbursements associated with Medicare Part D. In contrast, Tdap which is also covered under Medicare Part D was stocked much more than the zoster vaccine; it is possible that physicians did this because their patients thought they did not need a zoster vaccine or didn’t have adequate knowledge about the zoster vaccine or about the possibility to prevent shingles by vaccination. While financial barriers may play an important role in physicians’ decisions to routinely stock and subsequently recommend, administer or refer adult vaccines, there is a need for further research on barriers to and other factors that may affect adult vaccination decisions.^28^ Physicians commonly indicated a number of barriers on the patients’ side across all three vaccines: patients’ lack of motivation/willingness (they don’t think they need the vaccine) and concerns about potential adverse/side effects, as well as patients’ lack of knowledge/awareness about both the vaccine and the disease being prevented. This combined with the perception that there is not enough adequate information on adult vaccines for the patients seems to consistently indicate a need for more educational campaigns and materials designed for patients to further increase awareness about both the disease and the vaccine, highlighting risks and benefits. Finally, increasing physicians’ knowledge and awareness about vaccine insurance coverage may also help further reduce barriers to use of influenza, Tdap and zoster vaccines in adults in the US.

This survey-based study had a few limitations. Although efforts were made to ensure diverse geographical representation of physicians in the US, the surveyed physicians were not randomly sampled from the full US population of practicing GP/FP and internists, so results may not be generalizable broadly to this population. The response rate was 18.9% and the survey was taken during the physicians’ own time. Physicians were instructed to think about the past year when answering the survey questions, which may have introduced recall bias. In addition, it is possible that physicians may have reported what they should do compared to what they did in the past year.

In conclusion, this study reflected the need for further education of physicians in terms of ACIP recommendations and policy changes in insurance coverage which may help to increase the recommendation of adult vaccinations amongst eligible adult patients. Specifically, since physicians perceived more insurance-related barriers for patients with Medicare than commercial insurance, efforts to increase patient and physician knowledge or improve policies related to Medicare coverage could mitigate the differences seen in physician recommendation, administration, and referral for adult vaccines. Lastly, more efforts towards encouraging patients to attend wellness visits on a regular basis could potentially ignite an uptake in vaccination recommendations and administrations. Figure 6 summarizes the context, outcomes, and impact of this study for patients.

## Materials and methods

### Study design and set-up

A cross-sectional, internet-based survey was conducted in a convenience sample of US-based, board certified, GP/FP and IM physicians (N = 1,000). The study design was set-up in three phases (phase 1: pre-test interviews [N = 6]; phase 2: online pilot phase [N = 6] and phase 3: full administration [N = 1,000]).

Physicians were selected for each of the phases based on predefined eligibility criteria. They were included if their primary profession was either GP/FP or Internist; for FPs or Internists, they had to be board certified and included only if they had managed patients covered by both Medicare and commercial insurances. Physicians practicing in Vermont, Minnesota, and Massachusetts were excluded due to state-specific regulations, or if they had spent most of their time in treating patients in Government/Veterans Administration setting. Physicians who worked part-time or spent <60.0% of their time in clinical practice were excluded. Physicians who had <20.0% of patients fit into the age groups 19–64 years and ≥65 years or proportion of patients aged ≥19 years with commercial insurance <20.0% or proportion of patients aged ≥19 years with Medicare <10.0% were excluded. Finally, physicians from one phase were not allowed to participate in the other phases of the study.

Study materials (including protocol and draft surveys) and informed consent forms for physicians participating in all phases of the study were collected and submitted to Quorum Review Independent Review Board for review and approval prior to study implementation.

#### Phase 1: pre-test face-to-face interviews

The first draft of the survey was developed as a “paper and pencil” version based on the results from a review of the literature and the research team’s experience in conducting vaccination studies. Following survey development, qualitative face-to-face interviews were conducted with six physicians (three GP/FP and three internists) to gather feedback on the draft survey, designed to assess physician attitudes, knowledge, practices, and perceived barriers to adult vaccination against influenza, Tdap, and zoster. For the interviews, physicians who were practicing in the US, were selected using the M3 Global Research national panel (M3 Global Research is a database providing access to over two million physicians and one million HCPs globally) and verified using the American Medical Association (AMA) database.^29,30^ Interviews lasted up to 60 minutes and were conducted by an Adelphi Research moderator. Interview data were summarized, and results were used to inform revisions to the draft survey prior to phase 2.

Based on input from physicians during the interviews the draft survey was revised for use in phase 2.

#### Phase 2: online pilot testing via telephone interviews

In phase 2, the revised draft survey from phase 1 was programmed electronically for web-based administration. Telephone-based qualitative interviews were conducted with an additional six physicians meeting the same eligibility requirements as those in phase 1 to obtain feedback on a draft electronic version of the survey. Physicians were also selected from the M3 Global Research national panel. During this phase, physicians were selected from different geographical regions across the US: three physicians from the northeast, and one physician from each of the south, west, and mid-west regions. This selection of physicians was also verified using the AMA database. The web-based survey was shared with each physician online, and questions were asked to the physicians both during the completion of the survey, as well as at the end of the interview. Like phase 1, interviews lasted approximately 60 minutes after which interview data were summarized, and physician input from this phase was used to revise the survey prior to implementation in phase 3.

#### Phase 3: administration of the full survey

**Figure 7.**
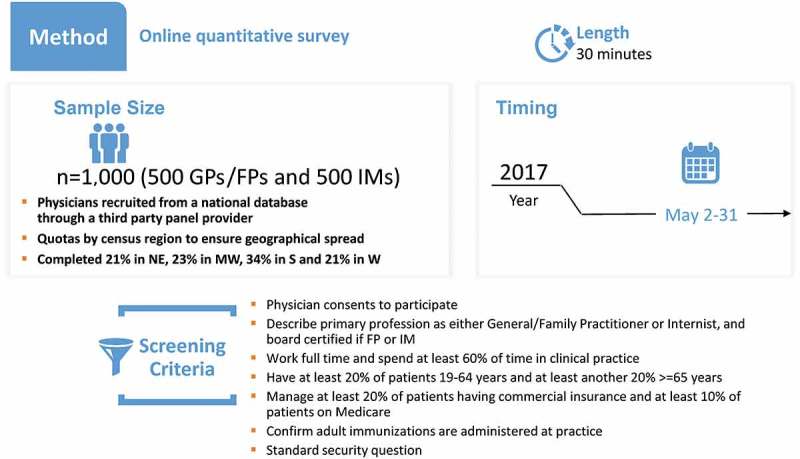
Survey design. FPs: family practice physicians; GPs: general practitioners; IMs: internal medicine physicians; NE: northeast; MW: midwest; S: south; W: west.

Conducted in May 2017, the survey resulting from phase 2 was administered to a convenience sample of 1,000 US-based physicians as the study targeted a total of 1,000 survey completions (Figure 7). Physicians in phase 3 were also chosen from the M3 Global Research national panel, and as in phases 1 and 2, the selection was verified using the AMA database. Initially, 5,100 GP/FP and 4,700 IM physicians were invited to participate in the study. A total of 1,852 physicians consented to participate (18.9% response rate). The survey was closed after the target of 1,000 was achieved; the study completion rate was 54.0%.

The final survey that was administered had a total of 80 questions, divided into two categories (the full survey is provided in Supplementary File 2). Category 1 had 66 questions which were used to assess the physicians’ attitudes, knowledge, self-reported practices and perceived barriers to adult influenza, Tdap and zoster vaccination. Category 2 had 14 questions which were used to gather information on physician and practice demographics. Physicians were asked to fill the survey based on the patients they treated during the previous year, who were 19 years or older and eligible for vaccination, according to the ACIP recommendations.

The online survey took approximately 30 minutes for completion. Following completion of the survey, each physician was compensated US$58 for time expended on the completion of survey.

### Analyses

No quantitative analyses were performed on the data collected from phases 1 and 2 of the study. Data for phase 3 of this study were collected using the Confirmit v22 software (Confirmit AS, London, UK), which is a market research data collection software. All analyses were done using Q statistical analysis v5.1.2 software (Research Software, Chicago, IL) and IBM SPSS Statistics v23 (IBM, Armonk, NY).

Descriptive statistics were used to summarize physician demographics, characteristics of patients seen by physicians, physician attitudes, knowledge, practices (recommendation, administration, and referral), and perceived barriers to adult vaccination. Results are presented as overall and by specific vaccine type (influenza, Tdap and zoster). Most variables were categorical, described by the frequency and percentage of each response choice. A limited number of variables were continuous, with corresponding means presented.

Univariate analysis/one-way analysis of variance (ANOVA) was used to determine statistically significant differences (p < 0.05) between two or more independent groups. Independent t-tests were used for means and chi-square tests for proportions.
