# Supplementary material for: Is patient insurance type related to physician recommendation, administration and referral for adult vaccination? A survey of US physicians
Source: Hum Vaccin Immunother. 2019 Mar 20;15(9):2217–26. doi: 10.1080/21645515.2019.1582402 (PMC6773384; doi:10.1080/21645515.2019.1582402)
Supplement: Supplemental Material [file khvi-15-09-1582402-s001.zip › Supplementary file 1 v4.docx]

**SUPPLEMENTARY FILE 1**

**Supplement Table 1. Physician-reported patient characteristics (N=1,000)**

| **Patient characteristic** | **Mean percentage, %** |
| --- | --- |
| **Age of patient** |  |
| Under 19 years or older | 12.4 |
| 19-64 years | 49.2 |
| 65 years and older | 38.4 |
| **Insurance coverage of adult patients seen in a typical year*** | |
| Commercial insurance | 55.8 |
| Medicare | 52.3 |
| **Insurance of patients 65 years and older** |  |
| Medicare | 39.9 |
| Medicare and commercial | 36.9 |
| Medicare advantage | 23.0 |
| **Primary insurance of patients 19 years or older** |  |
| Commercial | 49.2 |
| Medicare/Medicare advantage | 34.0 |
| Medicaid | 11.9 |
| Not covered/no insurance | 5.0 |

Note: Mean percentage of patients was calculated from all physician responses.

*Not mutually exclusive; of note, physicians were asked to report what percentage of their patients had certain diseases in the past year, to examine possible relationships with recommendation/administration/referral practices. Some incidences are higher than expected, possibly because of response fields utilized to answer the associated question or because physicians provided the percentage of patients who had received vaccinations for each disease.

**Supplement Figure 1. Level of Agreement with Statements Regarding Adult Vaccinations**


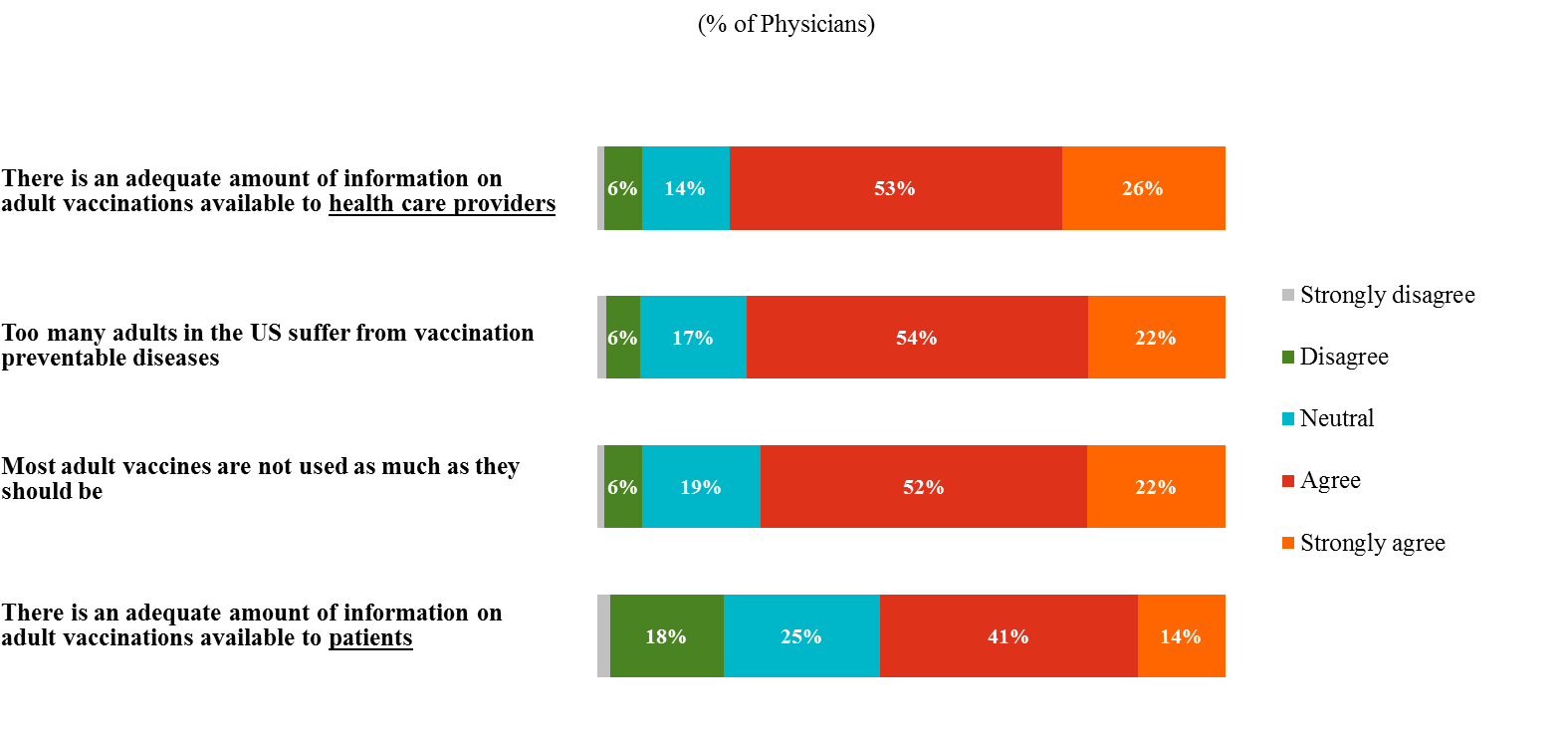


Data labels <5% not displayed; Total number of Physicians =1,000

Note: Sums of percentages may not equal 100% due to rounding.

**Supplement Figure 2. (A) General Knowledge of Advisory Committee on Immunization Practices (ACIP) Recommendations, (B) Physicians’ understanding of ACIP recommendations for receiving Influenza, Tdap or Zoster vaccination at the time of survey administration**


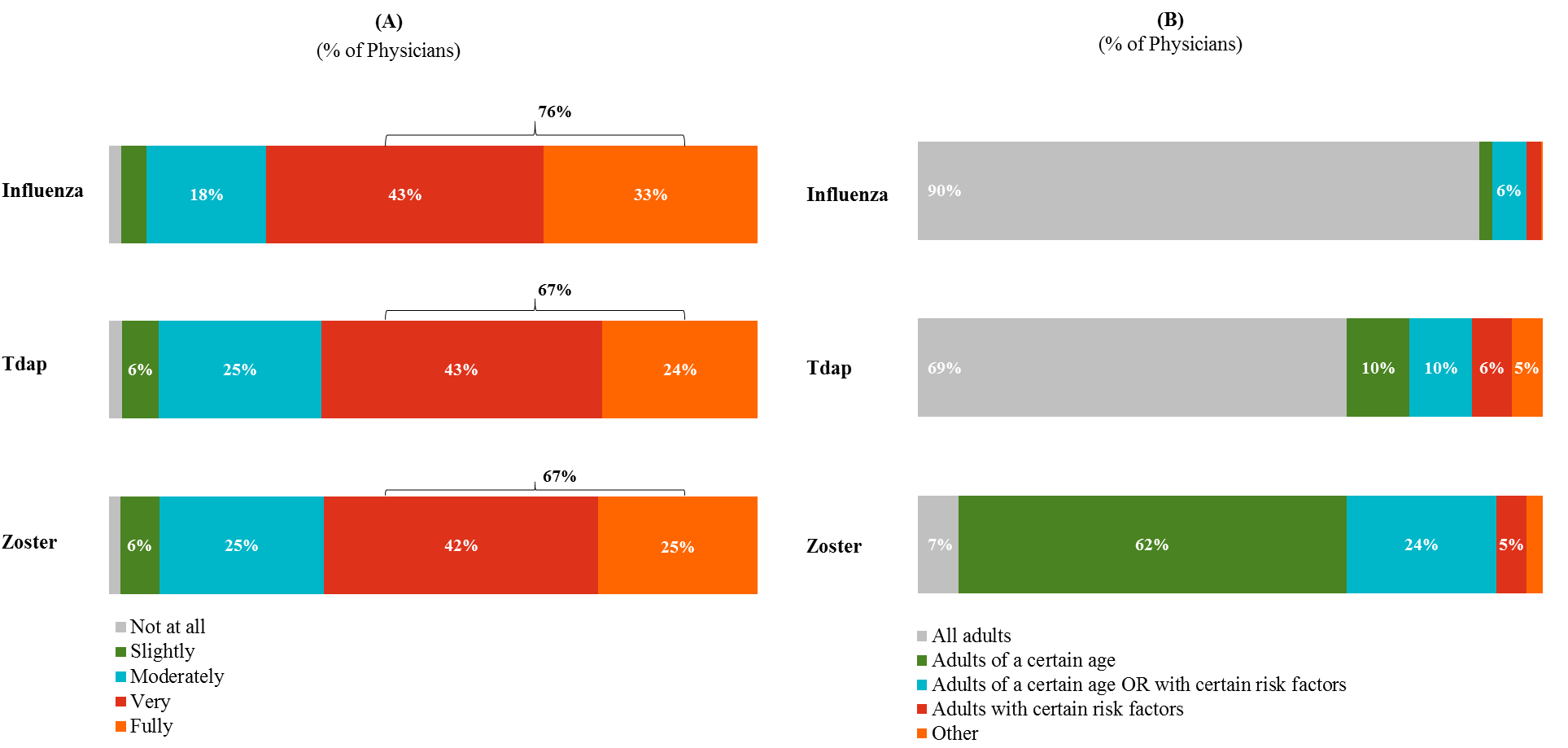


Total number of Physicians =1,000; Data labels <5% not displayed; Tdap, tetanus/diphtheria/acellular pertussis vaccine

Note: Sums of percentages may not equal 100% due to rounding.

**Supplement Figure 3. Physician knowledge of insurance coverage for adult vaccinations**


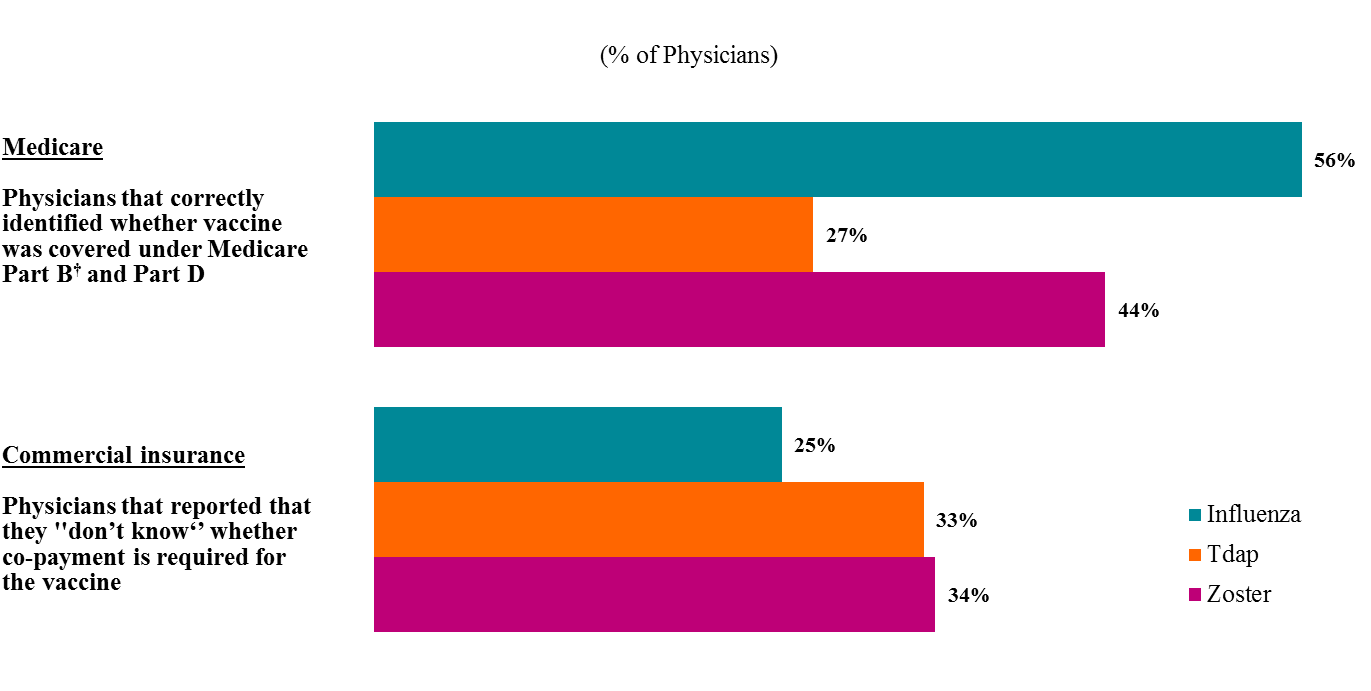


Total number of Physicians =1,000; ^†^Medicare Part B covers only the Influenza vaccine. Tdap and Zoster vaccines are covered under Medicare Part D; Tdap, tetanus/diphtheria/acellular pertussis vaccine

**Supplement Figure 4. Physician discussion of vaccination with eligible patients during patient visits (N=1,000)**


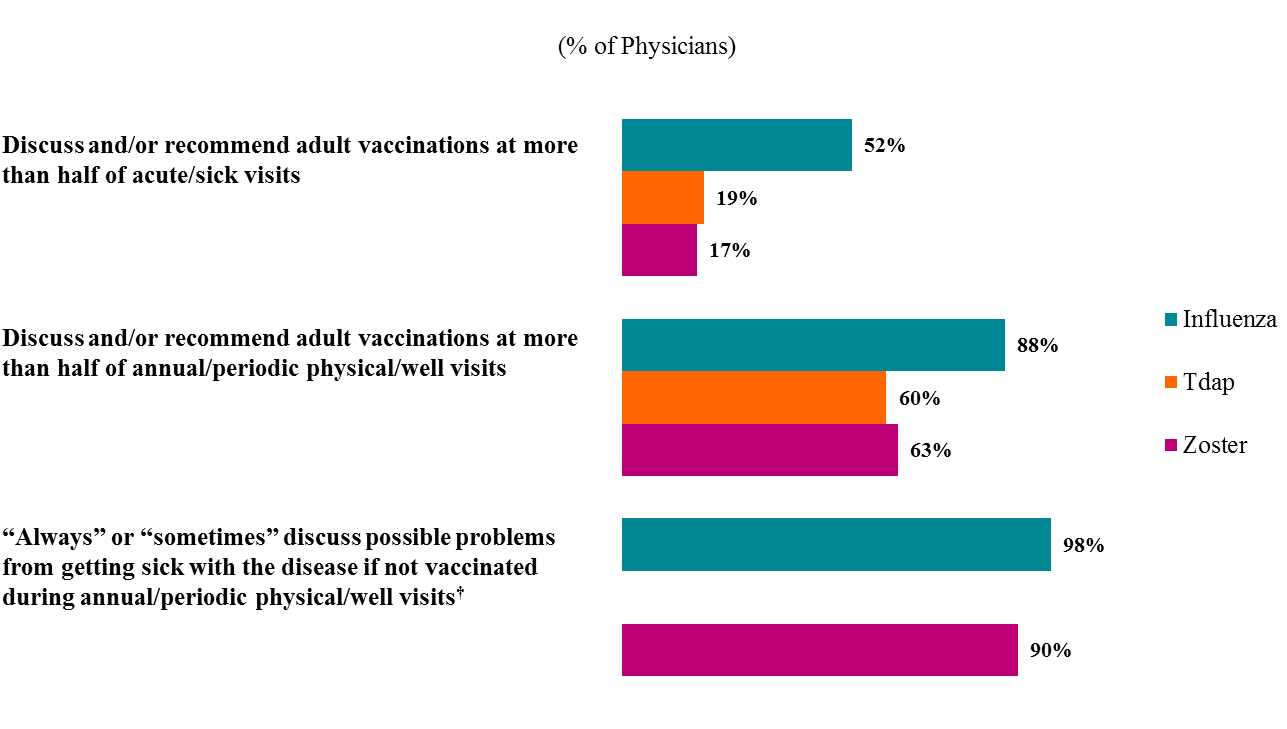


Total number of Physicians = 1,000; ^†^Data not shown for Tdap due to error in item wording; Tdap, tetanus/diphtheria/acellular pertussis vaccine

**Supplement Figure 5. (A) Level of Agreement with Statements Regarding Adult Vaccinations, (B) Physician report of stocking and referral of vaccines**


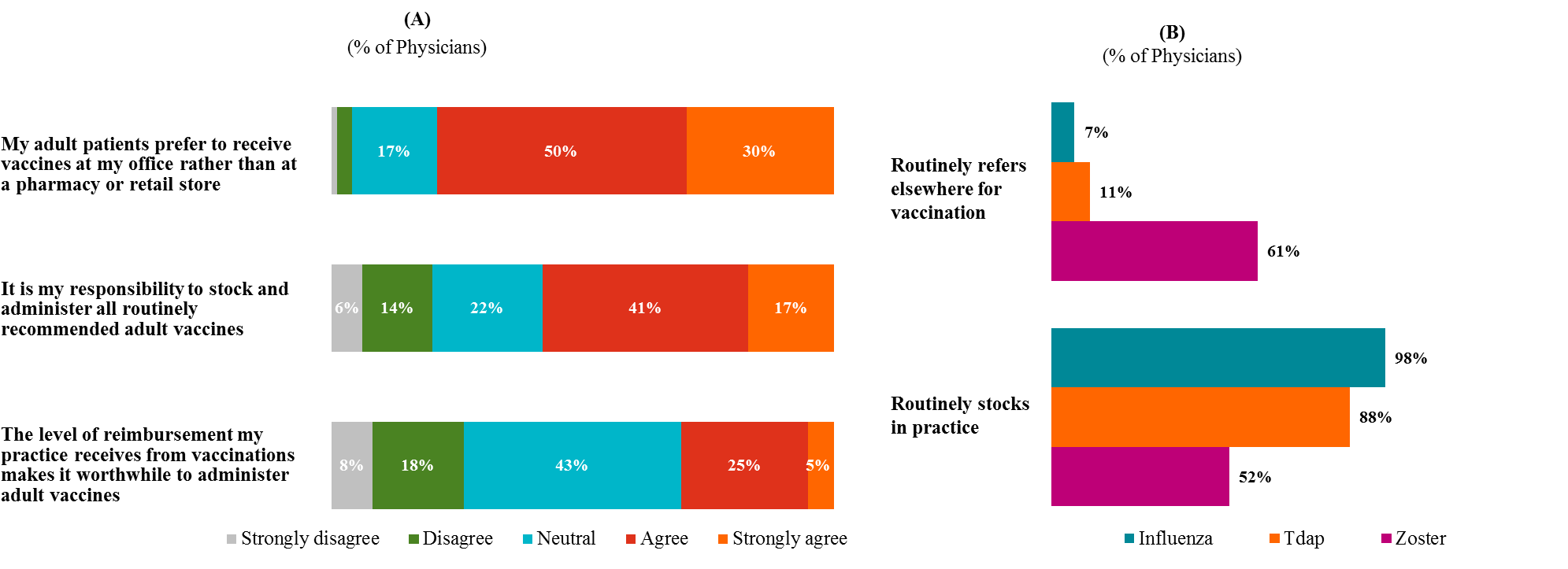


Data labels <5% not displayed; Total number of Physicians =1,000; Tdap, tetanus/diphtheria/acellular pertussis vaccine

Note: in (B), the categories are not mutually exclusive.

**Supplement Figure 6. Barriers to Eligible Adult Patients Receiving Each Vaccination**


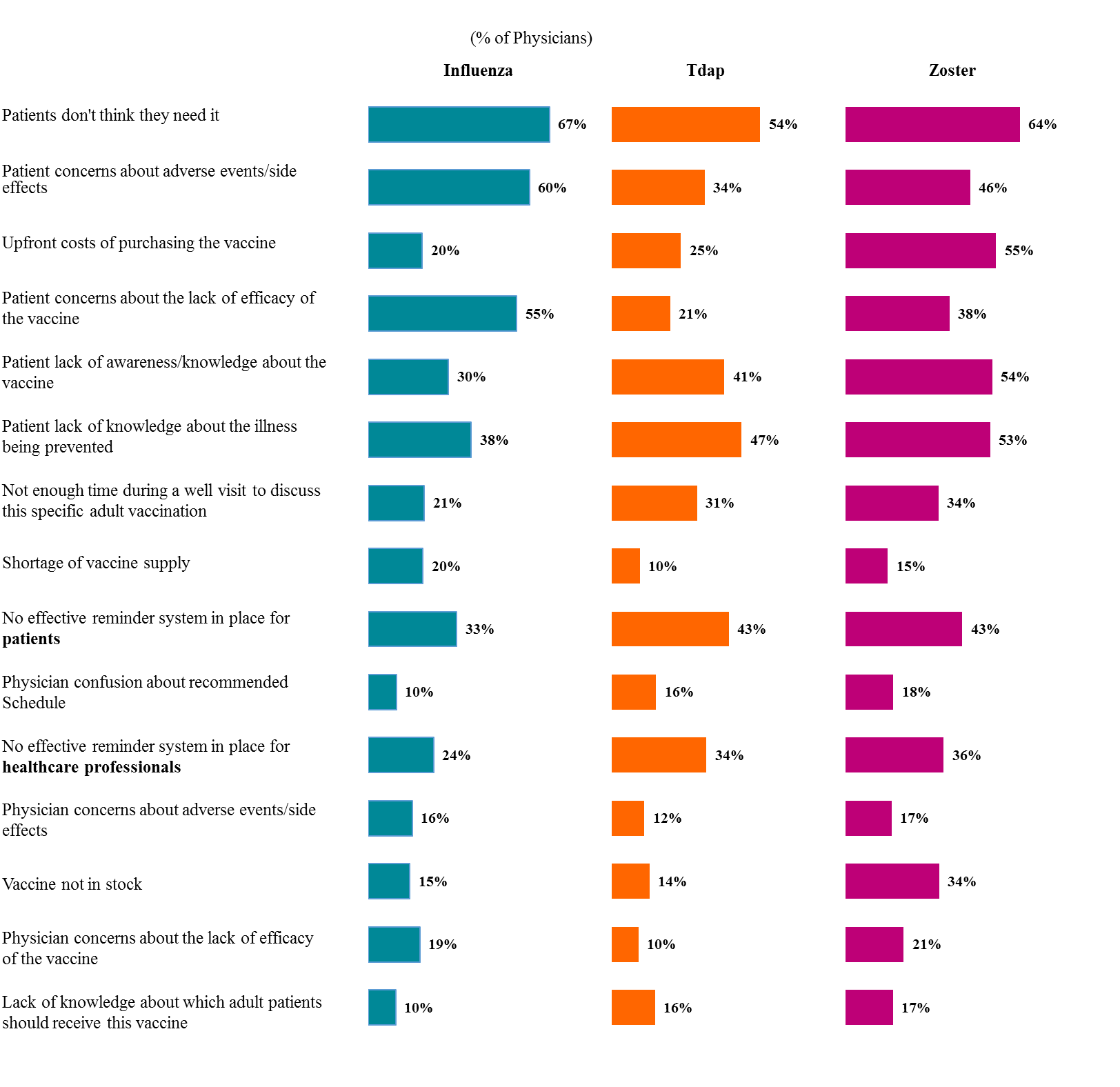


Total number of Physicians =1,000; Tdap, tetanus/diphtheria/acellular pertussis vaccine
