# Supplementary material for: Is patient insurance type related to physician recommendation, administration and referral for adult vaccination? A survey of US physicians
Source: Hum Vaccin Immunother. 2019 Mar 20;15(9):2217–26. doi: 10.1080/21645515.2019.1582402 (PMC6773384; doi:10.1080/21645515.2019.1582402)
Supplement: Supplemental Material [file khvi-15-09-1582402-s001.zip › Supplementary file 2.docx]

**SUPPLEMENTARY FILE 2**

**ONLINE SURVEY**

# Adverse Event Reporting

S1.

We are now required to pass on to our client details of adverse events that are raised during the course of research surveys. Although this is an online research survey and how you respond will, of course, be treated in confidence, should you raise an adverse event and / or product complaint, we will need to report this, even if it has already been reported by you directly to the company or the regulatory authority. The Adverse Event data collected may be sent outside the reporting country for processing.

We will initially forward the Adverse Report to the client’s pharmacovigilance department anonymously. Any requests for further information from pharmacovigilance will come through the recruiting agency.

|  | | | |
| --- | --- | --- | --- |
| **1** | 🔾 | I would like to proceed and give permission for the recruitment agency to contact me to provide more information about an adverse event mentioned by me during the survey. |  |
| **2** | 🔾 | I would like to proceed and **DO NOT** want the recruitment agency to contact me to provide more information about an adverse event mentioned by me during the survey |  |
| **3** | 🔾 | I don’t want to proceed | **🡺 TERMINATE** |

S2.

We may want to further explore your responses to some of the questions answered. Would you be prepared to be re-contacted by the agency that recruited you, to take part in additional research to clarify any responses that require further information?

|  | | |
| --- | --- | --- |
| **1** | 🔾 | Yes |
| **2** | 🔾 | No |

**S3-S4 OMITTED**

Thank you. First, I need to ask you a few questions to determine if you are eligible. S5.

Which of the following best describes your primary profession?

|  | | | |
| --- | --- | --- | --- |
| **1** | 🔾 | General Practitioner |  |
| **8** | 🔾 | Family Practitioner |  |
| **2** | 🔾 | Nurse Practitioner (NP) | **🡺 TERMINATE** |
| **3** | 🔾 | Internist |  |
| **4** | 🔾 | Geriatrician | **🡺 TERMINATE** |
| **5** | 🔾 | Physician Assistant (PA) | **🡺 TERMINATE** |
| **6** | 🔾 | Licensed Practical Nurse (LPN) | **🡺 TERMINATE** |
| **7** | 🔾 | Pediatrician | **🡺 TERMINATE** |
| **95** | 🔾 | Other (specify) | **🡺 TERMINATE** |

S6.

Are you currently board certified or board eligible in your primary specialty?

|  | | | |
| --- | --- | --- | --- |
| **1** | 🔾 | Board certified |  |
| **2** | 🔾 | Board eligible | **🡺 TERMINATE ONLY IF S5=8 OR S5=3** |
| **3** | 🔾 | Neither of the above | **🡺 TERMINATE ONLY IF S5=8 OR S5=3** |

S7.

How many years have you been in practice since completing your residency?

# years

# RANGE = 0-99

**ADELPHI TO MONITOR DURING FIELDING**

S7a.

Are you currently a government employee (federal, state, local)?

|  | | | |
| --- | --- | --- | --- |
| **1** | 🔾 | Yes | **🡺 CONTINUE TO S7B** |
| **2** | 🔾 | No | **🡺 SKIP TO S8** |

S7b.

Thank you for indicating that you are employed by the government. Regulations exist stating that an honorarium or enticement cannot be paid to government employees to participate in online research. Your opinion is valuable to us. Do you choose to continue and complete the survey without payment?

*Please click “Yes” if you would like to participate*

|  | | | |
| --- | --- | --- | --- |
| **1** | 🔾 | Yes | **🡺 CONTINUE** |
| **2** | 🔾 | No | **🡺 TERMINATE** |

S8.

Which state(s) are you licensed to practice in at this time?

## Select all that apply

**TERMINATE IF ANY OF THE FOLLOWING ARE SELECTED: VERMONT, MINNESOTA, AND MASSACHUSETTS QUOTA: CREATE VARIABLE FOR REGION IN ORDER TO ENSURE GEOGRAPHICAL REPRESENTATION**

**SET SOFT QUOTAS**

S8a.

Do you practice or otherwise work in a medical capacity in Vermont, even for only a limited time or any sort of regular frequency – for example, one day per year, or one week every three years?

|  | | | |
| --- | --- | --- | --- |
| **1** | 🔾 | Yes | **🡺 TERMINATE** |
| **2** | 🔾 | No | **🡺 CONTINUE** |

S9.

Which of the following best describes your practice?

|  | | | |
| --- | --- | --- | --- |
| **1** | 🔾 | Large Group Practice (5 or more doctors on staff) |  |
| **2** | 🔾 | Small Group Practice (2-4 doctors on staff) |  |
| **3** | 🔾 | Solo Practice (1 doctor on staff) |  |
| **95** | 🔾 | Other | **🡺 TERMINATE** |

S10.

In which of the following settings do you spend the majority of time treating patients?

|  | | | | **Community vs.**  **Academic** |
| --- | --- | --- | --- | --- |
| **1** | 🔾 | Private practice (located in office, clinic or hospital) |  |  |
| **2** | 🔾 | Outpatient clinic |  |  |
| **3** | 🔾 | Hospital: University or academic |  | **ACADEMIC** |
| **4** | 🔾 | Hospital: Community teaching |  | **COMMUNITY** |
| **5** | 🔾 | Hospital: Community non-teaching |  | **COMMUNITY** |
| **7** | 🔾 | Government/VA | **TERMINATE** |  |
| **95** | 🔾 | Other (specify) | **TERMINATE** |  |

# ASK IF PRIMARY SETTING IS NOT HOSPITAL (S10=1 OR 2)

S10a.

How would you describe your *primary* hospital affiliation?

|  | | |
| --- | --- | --- |
| **1** | 🔾 | University or academic hospital |
| **2** | 🔾 | Community teaching hospital |
| **3** | 🔾 | Community non-teaching hospital |

# ADELPHI TO MONITOR DURING FIELDING TO OBTAIN MIX

S11.

Which of the following BEST describes the location of your primary practice?

|  | | |
| --- | --- | --- |
| **1** | 🔾 | Urban |
| **2** | 🔾 | Suburban |
| **3** | 🔾 | Rural |

S12.

Do you currently work full-time or part-time at your practice?

|  | | | |
| --- | --- | --- | --- |
| **1** | 🔾 | Full-time |  |
| **2** | 🔾 | Part-time | **TERMINATE AFTER S15** |

S12a.

What percent of your professional time is spent in clinical practice as opposed to teaching, research, bench research, or administration?

% of time in clinical practice **🡺 TERMINATE IF < 60% AFTER S15 RANGE = 0-100**

S13a.

Approximately, how many patients do you personally see/treat in a **typical month** for any condition? *Do not include patients seen by colleagues.*

# patients seen in typical month

# RANGE: 0-9997

**TERMINATE HERE IF S13A=0**

S13b.

What proportion of all your patients fit into each of the following age groups?

## Your responses must total 100%.

| **RANGE FOR EACH: % of patients in 0–100 each age group** | | | |
| --- | --- | --- | --- |
| **1** | Under 19 years |  |  |
| **2** | 19-64 years |  | **TERMINATE IF <20% AFTER S15** |
| **3** | 65 years and older |  | **TERMINATE IF <20% AFTER S15** |
|  |  | **=100%** |  |

S14a.

Thinking of all your adult patients **aged 19 years or older**, what proportion of patients has each of the following as their **primary insurance**?

## Your responses must total 100%.

| **RANGE FOR EACH: 0–100 % of patients 19**  **years or older** | | | |
| --- | --- | --- | --- |
| **1** | Commercial | % | **TERMINATE IF <20% AFTER S15** |
| **2** | Medicare/Medicare Advantage | % | **TERMINATE IF <10% AFTER S15** |
| **3** | Medicaid | % |  |
| **4** | Not covered/no insurance | % |  |
|  |  | **=100%** |  |

S15.

Are adult immunizations (such as tetanus, influenza, pneumococcal) administered at your practice?

|  | | | |
| --- | --- | --- | --- |
| **1** | 🔾 | Yes |  |
| **2** | 🔾 | No | **🡺 ASK S15A** |

# ASK IF S15_2 & THEN TERMINATE

S15a.

Which of the following reasons prevent you from administering adult vaccines at your practice?

|  | | |
| --- | --- | --- |
| **1** | ❑ | Office infrastructure does not support vaccine administration |
| **2** | ❑ | Cost concerns for my patients |
| **3** | ❑ | Medicare reimbursement problems for my practice |
| **4** | ❑ | Lack of administrative reimbursement fees for Medicare patients |
| **5** | ❑ | I don’t believe adult vaccinations should be given in a doctor’s office |
| **6** | ❑ | I don’t think there is a need for adult vaccinations |
| **9** | ❑ | Other (please specify) |

S16.

Are you or any member of your household affiliated with a pharmaceutical company, healthcare company, or government agency as a *primary* clinical investigator, consultant, researcher, or in any other capacity?

|  | | | |
| --- | --- | --- | --- |
| **1** | 🔾 | Yes | **🡺 TERMINATE** |
| **2** | 🔾 | No |  |

**IF RESPONDENT QUALIFIES:** You are eligible to participate in this survey. Please click the >> button to begin the study.

**IF RESPONDENT TERMINATES:** At this time, you are not eligible to participate in this survey. Thank you very much for your interest.

# Section A: Recommendation & Knowledge

Throughout this survey we would like you to focus on your adult patients **aged 19 years or older.**

# NEW SCREEN

0.

Which of the following do you use to determine a patient’s **eligibility** for each of the following vaccines?

# Select all that apply for each column.

| **Influenza (during**  **RANDOMIZE VACCINES AND LIST months**  **[KEEP CODES 5 & 6 TOGETHER] surrounding**  **flu season)**  **(A)** | | | **Tetanus and diphtheria (Td)**  **(B)** | **Tetanus, diphtheria, pertussis (Tdap)**  **(C)** | **Zoster (shingles)**  **(D)** | **Pneumococcal 13-valent conjugate (PCV13)**  **(E)** | **Pneumococcal polysaccharide (PPSV23)**  **(F)** |
| --- | --- | --- | --- | --- | --- | --- | --- |
| **1** | Age | ❑ | ❑ | ❑ | ❑ | ❑ | ❑ |
| **2** | Co-morbidities/medical history | ❑ | ❑ | ❑ | ❑ | ❑ | ❑ |
| **3** | Patient occupation | ❑ | ❑ | ❑ | ❑ | ❑ | ❑ |
| **4** | Lifestyle factors | ❑ | ❑ | ❑ | ❑ | ❑ | ❑ |
| **5** | Vaccination history–documented | ❑ | ❑ | ❑ | ❑ | ❑ | ❑ |
| **6** | Vaccination history–patient stated/memory | ❑ | ❑ | ❑ | ❑ | ❑ | ❑ |
| **7** | Patient willingness/motivation | ❑ | ❑ | ❑ | ❑ | ❑ | ❑ |
| **8** | Insurance type | ❑ | ❑ | ❑ | ❑ | ❑ | ❑ |
| **95** | Other (specify)  **ALLOW FOR EACH VACCINE** | ❑ | ❑ | ❑ | ❑ | ❑ | ❑ |

**Q3 & Q7 MOVED EARLIER FOR SURVEY FLOW**

3.

How knowledgeable would you say you are about the **Advisory Committee on Immunization Practices (ACIP) recommendations** for each of the following vaccines?

| **RANDOMIZE VACCINES IN Not at all**  **SAME ORDER AS Q0 knowledgeable** | | | **Slightly knowledgeable** | **Moderately knowledgeable** | **Very knowledgeable** | **Fully knowledgeable** |
| --- | --- | --- | --- | --- | --- | --- |
| **1** | Influenza | 🔾 | 🔾 | 🔾 | 🔾 | 🔾 |
| **2** | Tetanus and diphtheria (Td) | 🔾 | 🔾 | 🔾 | 🔾 | 🔾 |
| **3** | Tetanus, diphtheria, pertussis (Tdap) | 🔾 | 🔾 | 🔾 | 🔾 | 🔾 |
| **4** | Zoster (shingles) | 🔾 | 🔾 | 🔾 | 🔾 | 🔾 |
| **5** | Pneumococcal 13-valent conjugate (PCV13) | 🔾 | 🔾 | 🔾 | 🔾 | 🔾 |
| **6** | Pneumococcal polysaccharide (PPSV23) | 🔾 | 🔾 | 🔾 | 🔾 | 🔾 |

1. **SHOW ‘OTHER’ TEXT BOX ONLY IF OTHER SELECTED**

To the best of your knowledge, what is the **ACIP recommendation** for each of the following vaccines?

| **RANDOMIZE IN SAME ORDER AS Q0** | | **Recommended for all adults**  (A) | **Recommended only for adults of a certain age**  (B) | **Recommended only for adults with certain risk factors** (C) | **Recommended for adults of a certain**  **age OR with Other**  **certain risk factors (specify):**  (D) (E) | | **No recommendation**  (F) | **Don’t know**  (G) |
| --- | --- | --- | --- | --- | --- | --- | --- | --- |
| **1** | Influenza | 🔾 | 🔾 | 🔾 | 🔾 | ❑ | 🔾 | 🔾 |
| **2** | Tetanus and diphtheria (Td) | 🔾 | 🔾 | 🔾 | 🔾 | ❑ | 🔾 | 🔾 |
| **3** | Tetanus, diphtheria, pertussis (Tdap) | 🔾 | 🔾 | 🔾 | 🔾 | ❑ | 🔾 | 🔾 |
| **4** | Zoster (shingles) | 🔾 | 🔾 | 🔾 | 🔾 | ❑ | 🔾 | 🔾 |
| **5** | Pneumococcal 13- valent conjugate (PCV13) | 🔾 | 🔾 | 🔾 | 🔾 | ❑ | 🔾 | 🔾 |
| **6** | Pneumococcal polysaccharide (PPSV23) | 🔾 | 🔾 | 🔾 | 🔾 | ❑ | 🔾 | 🔾 |

# NEW SCREEN

**SHOW LINK FOR ELIGIBLE DEFINITION FOR THE FOLLOWING QUESTIONS: 1, 8, 8A, 10, 10B, 11, 11A, 11B, 12C, 12D, 12E, 14, 17A, 17B, 18B_1, 18B_2, 22, 22A, 29, 36, 36A, 38, 54, 60, 60A**

When we refer to adult patients **eligible** for vaccines, please consider the following definition based on the

**Advisory Committee on Immunization Practices (ACIP)** recommendations.

**Eligible** = Adults who meet guideline criteria for ACIP recommendations regarding:

- - Age requirement
  - Lack documentation of vaccination
  - Lack evidence of past infection
  - Additional medical conditions or other indications

**Do not consider insurance coverage**

A link back to this definition is provided throughout the survey for your reference

1.

Thinking about the past year, which of the following vaccines **do you routinely recommend, *not necessarily prescribe/administer*, in your practice** to **eligible** adult patients ages 19 years or older?

## Select all that apply

| **RANDOMIZE (KEEP CODES 2 & 3 TOGETHER AND CODES 5 & 6 TOGETHER)** | | |
| --- | --- | --- |
| **1** | Influenza (during months surrounding flu season) | ❑ |
| **2** | Tetanus and diphtheria (Td) | ❑ |
| **3** | Tetanus, diphtheria, pertussis (Tdap) | ❑ |
| **4** | Zoster (shingles) | ❑ |
| **5** | Pneumococcal 13-valent conjugate (PCV13) | ❑ |
| **6** | Pneumococcal polysaccharide (PPSV23) | ❑ |
| **96** | None of the above **MUTUALLY EXCLUSIVE** | 🔾 |

2.

Which of the following vaccines do you routinely **stock** at your practice/hospital?

## Select all that apply

| **RANDOMIZE IN SAME ORDER AS Q1** | | |
| --- | --- | --- |
| **1** | Influenza (during months surrounding flu season) | ❑ |
| **2** | Tetanus and diphtheria (Td) | ❑ |
| **3** | Tetanus, diphtheria, pertussis (Tdap) | ❑ |
| **4** | Zoster (shingles) | ❑ |
| **5** | Pneumococcal 13-valent conjugate (PCV13) | ❑ |
| **6** | Pneumococcal polysaccharide (PPSV23) | ❑ |
| **96** | None of the above **MUTUALLY EXCLUSIVE** | 🔾 |

**2A OMITTED**

2b.

For which of the following vaccines do you routinely **refer** patients elsewhere (i.e., another clinic or pharmacy; this may include writing a prescription) for the vaccine? ***Select all that apply***

| **RANDOMIZE IN SAME ORDER AS Q1** | | |
| --- | --- | --- |
| **1** | Influenza (during months surrounding flu season) | ❑ |
| **2** | Tetanus and diphtheria (Td) | ❑ |
| **3** | Tetanus, diphtheria, pertussis (Tdap) | ❑ |
| **4** | Zoster (shingles) | ❑ |
| **5** | Pneumococcal 13-valent conjugate (PCV13) | ❑ |
| **6** | Pneumococcal polysaccharide (PPSV23) | ❑ |
| **96** | None of the above **MUTUALLY EXCLUSIVE** | 🔾 |

**Q3 & Q7 MOVED EARLIER**

- 1. **OMITTED**

# SPLIT ON TWO SEPARATE SCREENS

7a.

To what extent do you agree or disagree with each of the following statements that can be made about adult vaccinations?

| **RANDOMIZE (KEEP 9 & 10 TOGETHER) Strongly**  **disagree** | | | **Disagree** | **Neutral** | **Agree** | **Strongly agree** |
| --- | --- | --- | --- | --- | --- | --- |
| **1** | Most adult vaccines are not used as much as they should be | 🔾 | 🔾 | 🔾 | 🔾 | 🔾 |
| **2** | Too many adults in the US suffer from vaccination preventable diseases | 🔾 | 🔾 | 🔾 | 🔾 | 🔾 |
| **4** | It is my responsibility to ensure my patients receive the recommended vaccinations, even if they get them somewhere else | 🔾 | 🔾 | 🔾 | 🔾 | 🔾 |
| **5** | I actively encourage my adult patients to keep track of their own vaccination schedule | 🔾 | 🔾 | 🔾 | 🔾 | 🔾 |
| **6** | I rely on my nurses/office staff to make recommendations/remind patients about adult vaccinations | 🔾 | 🔾 | 🔾 | 🔾 | 🔾 |
| **7** | The level of reimbursement my practice receives from vaccinations makes it worthwhile to administer adult vaccines | 🔾 | 🔾 | 🔾 | 🔾 | 🔾 |
| **8** | I actively keep track of my adult patients’ vaccination status/schedule in order to discuss during routine and/or well visits | 🔾 | 🔾 | 🔾 | 🔾 | 🔾 |
| **9** | There is an adequate amount of information on adult vaccinations available to healthcare providers | 🔾 | 🔾 | 🔾 | 🔾 | 🔾 |
| **10** | There is an adequate amount of information on adult vaccinations available to patients | 🔾 | 🔾 | 🔾 | 🔾 | 🔾 |
| **11** | It is my responsibility to stock and administer all routinely recommended adult vaccines | 🔾 | 🔾 | 🔾 | 🔾 | 🔾 |
| **12** | My adult patients prefer to receive vaccines at my office rather than at a pharmacy or retail store | 🔾 | 🔾 | 🔾 | 🔾 | 🔾 |

8.

To what extent is each of the following a **barrier** to **eligible (i.e. meet guideline criteria for ACIP recommendation)** adult patients receiving vaccinations?

| **To what extent is each a barrier:** | | | | | |
| --- | --- | --- | --- | --- | --- |
| **Not at all**  **RANDOMIZE** (A) | | | **Minor**  (B) | **Moderate**  (C) | **Major**  (D) |
| **1** | Patients not having regular well visits/health checks | 🔾 | 🔾 | 🔾 | 🔾 |
| **2** | Not enough time during the visit to discuss adult vaccinations | 🔾 | 🔾 | 🔾 | 🔾 |
| **3** | No record of when/if the patient received a particular vaccination | 🔾 | 🔾 | 🔾 | 🔾 |
| **4** | Patient fear of needles | 🔾 | 🔾 | 🔾 | 🔾 |

8a.

You indicated that all of the following represent a barrier to eligible adult patients to receiving vaccinations. Please rank the top 3 barriers, i.e. the ones that prevent the largest numbers of patients to be vaccinated.

## The largest barrier should be ranked “1”, the next largest barrier should be ranked “2”, and the next largest barrier should be ranked “3”

| 1. |
| --- |
| 2. |
| 3. |

IF 3 OR MORE OPTIONS = D AT Q8, THEN ONLY SHOW THOSE OPTIONS IF LESS THAN 3 = D, SHOW ALL OPTIONS = C AND D IF LESS THAN 3 = C AND D, SHOW ALL OPTIONS RATED B, C, D **NEVER SHOW ANY OPTIONS = A**

# Section B: Perceptions of Adult Vaccinations

Now we would like to understand more about your patients and your views of adult vaccinations. [INCLUDE VARIABLE IN PORTAL FOR COMMERCIAL AND MEDICARE ROTATION]

9.

Approximately what percent of your adult patients with the following as their primary insurance have an annual or periodic physical/well visit?

***Your responses do not need to equal 100%.***

# SHOW ONE COLUMN AT A TIME

| **ROTATE COMMERCIAL AND MEDICARE**  **Commercial**  **IN SAME ORDER THROUGHOUT SURVEY** | | | **Medicare** |
| --- | --- | --- | --- |
| **1** | % of adult patients that have annual or periodic physical/well visits | % | % |

**RANGE 0-100 FOR EACH CELL**

10.

Considering the time you have to spend with a patient during **an annual/periodic physical/well visit**, and all of the topics you have to cover, how many of these visits do you **discuss and/or make recommendations** for each of the following adult vaccinations to your **eligible** adult patients?

***Please select one per row.***

| **0-25%**  **RANDOMIZE IN SAME ORDER AS Q1**  **of visits** | | | **26-50%**  **of visits** | **51-75%**  **of visits** | **76-100%**  **of visits** |
| --- | --- | --- | --- | --- | --- |
| **1** | Influenza (during months surrounding flu season) | 🔾 | 🔾 | 🔾 | 🔾 |
| **2** | Tetanus and diphtheria (Td) | 🔾 | 🔾 | 🔾 | 🔾 |
| **3** | Tetanus, diphtheria, pertussis (Tdap) | 🔾 | 🔾 | 🔾 | 🔾 |
| **4** | Zoster (shingles) | 🔾 | 🔾 | 🔾 | 🔾 |

10b.

Which of the following vaccinations, if any, do you ever discuss and/or make recommendations for during an acute/sick visit to your **eligible** adult patients?

| **RANDOMIZE IN SAME ORDER AS Q1** | | |
| --- | --- | --- |
| **1** | Influenza (during months surrounding flu season) | ❑ |
| **2** | Tetanus and diphtheria (Td) | ❑ |
| **3** | Tetanus, diphtheria, pertussis (Tdap) | ❑ |
| **4** | Zoster (shingles) | ❑ |
| **96** | None of the above **MUTUALLY EXCLUSIVE** | 🔾 |

# SKIP IF Q10B=96, OTHERWISE SHOW EACH VACCINE SELECTED IN Q10B

11.

And **during an acute/sick event visit**, again given the limited time you have available with an individual patient, how many of these visits do you typically **discuss and/or make recommendations** to your **eligible** adult patients for each of the following adult vaccinations?

***Please select one per row.***

| **1-25%**  **RANDOMIZE IN SAME ORDER AS Q1**  **of visits** | | | **26-50%**  **of visits** | **51-75%**  **of visits** | **76-100%**  **of visits** |
| --- | --- | --- | --- | --- | --- |
| **1** | Influenza (during months surrounding flu season) | 🔾 | 🔾 | 🔾 | 🔾 |
| **2** | Tetanus and diphtheria (Td) | 🔾 | 🔾 | 🔾 | 🔾 |
| **3** | Tetanus, diphtheria, pertussis (Tdap) | 🔾 | 🔾 | 🔾 | 🔾 |
| **4** | Zoster (shingles) | 🔾 | 🔾 | 🔾 | 🔾 |

# NEW SCREEN

Now please think about your adult patients that are **eligible (i.e. meet guideline criteria for ACIP recommendation)** to receive the following vaccinations:

- - - Zoster (shingles)
    - Tetanus, diphtheria, pertussis (Tdap)
    - Influenza (during months surrounding flu season)

# NEW SCREEN

**Q17A-Q18B_2 MOVED BEFORE Q11A AND REVISED TO SHOW 3 VACCINES**

17a.

Thinking of your adult patients aged 19 years or older seen in the past year, approximately what percent do you believe are **eligible** to receive each of the following vaccines **(i.e. meet guideline criteria for ACIP recommendation)**?

## Enter a number between 0-100% for each vaccine

**SHOW ONE COLUMN AT A TIME AND BUILD UNTIL THREE SHOWN; IF TOTAL=100%, SHOW ERROR MESSAGE:**

**Your responses do not need to add to 100%. They may add to more than 100% as some patients are eligible for more than one vaccine.**

| **DO NOT SHOW Q#S** | | **Q17A** | **Q30A** | **Q55A** |
| --- | --- | --- | --- | --- |
| **RANDOMIZE**  **RANGE 0-100 FOR EACH CELL** | | **Zoster (shingles)** | **Tetanus, diphtheria, pertussis (Tdap)** | **Influenza (during months surrounding flu season)** |
| **1** | **% of Adult Patients Eligible for each Vaccine (in past year)** | % | % | % |

**ROTATE COMMERCIAL AND MEDICARE IN SAME ORDER THROUGHOUT SURVEY**

17b.

Now thinking of your adult patients that you have seen in the past year and are **eligible** to receive the following vaccines, to approximately what percent of these patients with each of the following types of insurance have you ever **recommended** the vaccines to?

Recommendation includes **actually administering** the vaccine to the patient, **and prescribing** or **referring** the patient to another clinic or pharmacy for the vaccine, as well as those for whom you recommended but the patient refused it.

Please take into account the time you had available with these patients, if any, to discuss vaccines.

## Your total does not need to equal 100% for each vaccine.

**SHOW ONE COLUMN AT A TIME; DO NOT ALLOW CHANGES TO THE 1ST COLUMN SHOWN**

|  | **RANDOMIZE IN SAME ORDER Commercial AS Q17A Insurance** | | | **Medicare** |
| --- | --- | --- | --- | --- |
| **SHOW Q17B IF Q17A>0** | **1** | Zoster (shingles) | % | % |
| **SHOW Q30B IF Q30A>0** | **2** | Tetanus, diphtheria, pertussis (Tdap) | % | % |
| **SHOW Q55B IF Q55A>0** | **4** | Influenza (during months surrounding flu season) | % | % |

**SHOW Q18B_1 AND Q18B_2 ON SAME SCREEN**

18b_1.

For approximately what percent of your **eligible** adult patients with **Commercial Insurance** have you or your office staff actually administered or referred for each of the following vaccines in the past year?

## Your total must equal 100% for each vaccine.

**ROTATE COMMERCIAL AND MEDICARE IN SAME ORDER THROUGHOUT SURVEY**

| **DO NOT SHOW Q#S** | | **Q18B_1** | **Q31B_1** | **Q57B_1** |
| --- | --- | --- | --- | --- |
| **RANDOMIZE IN SAME ORDER AS Q17A**  **RANGE 0-100 FOR EACH CELL** | | **Zoster (shingles)** | **Tetanus, diphtheria, pertussis (Tdap)** | **Influenza (during months surrounding flu season)** |
|  | **% of Eligible Patients with Commercial Insurance:** |  |  |  |
| **1** | Administered | % | % | % |
| **2** | Referred to another clinic or pharmacy | % | % | % |
| **3** | Neither | % | % | % |
|  | **TOTAL** | **=100%** | **=100%** | **=100%** |

18b_2.

For approximately what percent of your **eligible** adult patients with **Medicare** have you or your office staff actually administered or referred for each of the following vaccines in the past year?

## Your total must equal 100% for each vaccine.

| **DO NOT SHOW Q#S** | | **Q18B_2** | **Q31B_2** | **Q57B_2** |
| --- | --- | --- | --- | --- |
| **RANDOMIZE IN SAME ORDER AS Q17A**  **RANGE 0-100 FOR EACH CELL** | | **Zoster (shingles)** | **Tetanus, diphtheria, pertussis (Tdap)** | **Influenza (during months surrounding flu season)** |
|  | **% of Eligible Patients with Medicare:** |  |  |  |
| **1** | Administered | % | % | % |
| **2** | Referred to another clinic or pharmacy | % | % | % |
| **3** | Neither | % | % | % |
|  | **TOTAL** | **=100%** | **=100%** | **=100%** |

11a.

To what extent is each of the following a **barrier** to eligible adult patients receiving each vaccination?

# PROGRAMMER: SHOW ONE BARRIER PER SCREEN

| **RANDOMIZE ORDER OF BARRIERS** | |
| --- | --- |
| **6** | Not enough time during a well visit to discuss this specific adult vaccination |
| **7** | No effective reminder system in place for healthcare professionals |
| **8** | No effective reminder system in place for patients |
| **9** | Patient lack of knowledge about the illness being prevented |
| **10** | Patient lack of awareness/knowledge about the vaccine |
| **11** | Patients don’t think they need it |
| **12** | Patient concerns about adverse events/side effects |
| **13** | Patient concerns about the lack of efficacy of the vaccine |
| **14** | Physician concerns about adverse events/side effects |
| **15** | Physician concerns about the lack of efficacy of the vaccine |
| **16** | Physician lack of knowledge about which adult patients should receive this vaccine |
| **17** | Vaccine not in stock |
| **18** | Shortage of vaccine supply |
| **19** | Upfront costs to my practice of purchasing the vaccine |
| **20** | Physician confusion about recommended schedule |

**Not enough time during a well visit to discuss this specific adult vaccination**

| **To what extent is the above item a barrier for each vaccine:** | | | | | |
| --- | --- | --- | --- | --- | --- |
| **Not at all**  **RANDOMIZE VACCINES IN SAME** (A)  **ORDER FOR EACH BARRIER** | | | **Minor**  (B) | **Moderate**  (C) | **Major**  (D) |
| **1** | Zoster (shingles) | 🔾 | 🔾 | 🔾 | 🔾 |
| **2** | Tetanus, diphtheria, pertussis (Tdap) | 🔾 | 🔾 | 🔾 | 🔾 |
| **4** | Influenza (during months surrounding flu season) | 🔾 | 🔾 | 🔾 | 🔾 |

11b.

You indicated that all of the following represent a barrier to eligible adult patients receiving the [**INSERT VACCINATION RANDOMIZED FIRST**] vaccine. Please rank up to 5 barriers, i.e. the ones that prevent the largest numbers of patients to be vaccinated.

## The largest barrier should be ranked “1”, the next largest barrier should be ranked “2”, the next largest barrier should be ranked “3” and so on up to “5”.

*Please scroll down to review the entire list, as necessary.*

| 1. |
| --- |
| 2. |
| 3. |
| 4. |
| 5. |

IF 5 OR MORE OPTIONS = D AT Q11A, THEN ONLY SHOW THOSE OPTIONS IF LESS THAN 5 = D, SHOW ALL OPTIONS = C AND D IF LESS THAN 5 = C AND D, SHOW ALL OPTIONS RATED B, C, D **NEVER SHOW ANY OPTIONS = A**

**IF MORE THAN 5 SHOWN, DO NOT ALLOW MORE THAN 5 TO BE RANKED**

# REPEAT RANKING EXERCISE FOR REMAINING VACCINES IN ORDER OF RANDOMIZATION

12a.

Thinking of your patients on **Medicare**, what is the **typical market access coverage** for each of the following vaccines?

| **RANDOMIZE IN SAME ORDER AS Q1** | | **Covered by Medicare Part B** | **Covered by Medicare Part D** | **Don’t know** |
| --- | --- | --- | --- | --- |
| **1** | Influenza | 🔾 | 🔾 | 🔾 |
| **2** | Tetanus and diphtheria (Td) | 🔾 | 🔾 | 🔾 |
| **3** | Tetanus, diphtheria, pertussis (Tdap) | 🔾 | 🔾 | 🔾 |
| **4** | Zoster (shingles) | 🔾 | 🔾 | 🔾 |

12b.

Thinking of your patients with **commercial insurance**, do each of the following vaccines typically require a

# patient co-pay?

*When thinking of the patient co-pay, please answer in regards to the* ***vaccine****,* ***not*** *the office visit.*

| **RANDOMIZE IN SAME ORDER AS Q1 Requires Co-**  **pay** | | | **Does NOT Require co-Pay** | **Don’t know** |
| --- | --- | --- | --- | --- |
| **1** | Influenza | 🔾 | 🔾 | 🔾 |
| **2** | Tetanus and diphtheria (Td) | 🔾 | 🔾 | 🔾 |
| **3** | Tetanus, diphtheria, pertussis (Tdap) | 🔾 | 🔾 | 🔾 |
| **4** | Zoster (shingles) | 🔾 | 🔾 | 🔾 |

**Q12C. SHOW COMMERCIAL INSURANCE & MEDICARE ON SAME SCREEN; SHOW ONE BARRIER PER SCREEN**

| **RANDOMIZE ORDER OF BARRIERS** | |
| --- | --- |
| **21** | Vaccine not being covered by patient insurance |
| **22** | Patient out of pocket cost / copay being too high |
| **23** | Reimbursement process for my practice (i.e., billing system) |
| **24** | Lack of adequate reimbursement to my practice for vaccine purchase |
| **25** | Lack of adequate reimbursement to my practice for vaccine administration |
| **26** | Lack of physician knowledge whether the vaccine is covered by the patient’s insurance |

12c.

Now please think about your adult patients with **[**Commercial Insurance / Medicare**]** and your adult patients with **[**Medicare / Commercial Insurance**]**. **RANDOMIZE ORDER IN INTRO SENTENCE, ANSWER GRIDS & RANKING EXERCISE WITH INSURANCE TYPE IN SAME ORDER THROUGHOUT SURVEY**

To what extent is the following a **barrier** to eligible adult patients with each insurance type for the following vaccinations:

**Vaccine not being covered by patient insurance**

|  |  | **For patients with Commercial Insurance** | | | |
| --- | --- | --- | --- | --- | --- |
|  |  | **(A)**  **Not at all** | **(B)**  **Minor** | **(C)**  **Moderate** | **(D)**  **Major** |
| **1** | Zoster (shingles) | 🔾 | 🔾 | 🔾 | 🔾 |
| **2** | Tetanus, diphtheria, pertussis (Tdap) | 🔾 | 🔾 | 🔾 | 🔾 |
| **4** | Influenza (during months surrounding flu season) | 🔾 | 🔾 | 🔾 | 🔾 |

| **For patients with Medicare** | | | |
| --- | --- | --- | --- |
| **(A)**  **Not at all** | **(B)**  **Minor** | **(C)**  **Moderate** | **(D)**  **Major** |
| 🔾 | 🔾 | 🔾 | 🔾 |
| 🔾 | 🔾 | 🔾 | 🔾 |
| 🔾 | 🔾 | 🔾 | 🔾 |

12d/e.

For each type of insurance, please rank the top 3 barriers, i.e. the ones that prevent the largest numbers of

**eligible** adult patients to be vaccinated with [**INSERT VACCINATION RANDOMIZED FIRST IN 12C**].

## The largest barrier should be ranked “1”, the next largest barrier should be ranked “2”, and the next largest barrier should be ranked “3”

| **DO NOT SHOW Q#S** | | **Q12D** | **Q12E** |
| --- | --- | --- | --- |
| **RANDOMIZE BARRIERS IN SAME ORDER AS Q12C; RANDOMIZE COMMERCIAL AND INSURANCE IN SAME ORDER AS PRIOR QUESTIONS** | | **Commercial Medicare Insurance** | |
| **21** | Vaccine not being covered by patient insurance |  |  |
| **22** | Patient out of pocket cost / copay being too high |  |  |
| **23** | Reimbursement process for my practice (i.e., billing system) |  |  |
| **24** | Lack of adequate reimbursement to my practice for vaccine purchase |  |  |
| **25** | Lack of adequate reimbursement to my practice for vaccine administration |  |  |
| **26** | Lack of physician knowledge whether the vaccine is covered by the patient’s insurance |  |  |

**REPEAT RANKING EXERCISE FOR REMAINING VACCINES IN ORDER OF RANDOMIZATION**

**ROTATE SECTIONS C AND D AND F [INCLUDE VARIABLE IN PORTAL FOR ROTATION]**

**Section C: Zoster Vaccination**

Now we would like to focus specifically on the **Zoster (shingles) vaccine.**

13.

How knowledgeable would you say you are about the following aspects of the **Zoster (shingles) vaccine**?

| **RANDOMIZE Not at all knowledgeable** | | | **Slightly knowledgeable** | **Moderately knowledgeable** | **Very knowledgeable** | **Fully knowledgeable** |
| --- | --- | --- | --- | --- | --- | --- |
| **1** | Commercial market access coverage | 🔾 | 🔾 | 🔾 | 🔾 | 🔾 |
| **2** | Medicare market access coverage | 🔾 | 🔾 | 🔾 | 🔾 | 🔾 |
| **3** | Which adult patients should receive it | 🔾 | 🔾 | 🔾 | 🔾 | 🔾 |

14.

To which of the following groups of **eligible** adult patients do you routinely recommend the **Zoster (shingles) vaccine**?

|  | | |
| --- | --- | --- |
| **1** | All adults/everybody  **[MUTUALLY EXCLUSIVE]** | 🔾 |
| **98** | None – I don’t recommend this vaccine to adult patients **[MUTUALLY EXCLUSIVE]** | 🔾 |
| **HEADER** | **Depends (select all that apply below)** |  |
| **2** | Patients of a certain age | ❑ |
| **3** | Patients with certain risk factors | ❑ |
| **95** | Other (please specify) | ❑ |

# ASK IF Q14_2 SELECTED

15.

To which of the following age groups do you routinely recommend a **Zoster (shingles) vaccine** if no known risk factors?

## Select all that apply

|  | | |
| --- | --- | --- |
| **1** | Ages 19-29 | ❑ |
| **2** | Ages 30-39 | ❑ |
| **3** | Ages 40-49 | ❑ |
| **4** | Ages 50-59 | ❑ |
| **5** | Ages 60-64 | ❑ |
| **6** | Ages 65 and older | ❑ |

**ASK IF Q14_3 SELECTED**

16.

You said you recommend a **Zoster (shingles) vaccine** to patients with **certain risk factors**. To which of the following patient types do you recommend a Zoster (shingles) vaccine? ***Select all that apply***

| **RANDOMIZE** | | |
| --- | --- | --- |
| **1** | Pregnant patients | ❑ |
| **2** | Immuno-compromised conditions | ❑ |
| **3** | Men who have sex with other men | ❑ |
| **4** | Kidney failure / end-stage renal disease / dialysis | ❑ |
| **5** | Heart disease | ❑ |
| **6** | Chronic lung disease (e.g. asthma, COPD) | ❑ |
| **7** | Chronic liver disease (e.g. Hepatitis) | ❑ |
| **8** | Chronic alcoholism | ❑ |
| **9** | Asplenia (non-functioning spleen) | ❑ |

| **10** | Diabetics | ❑ |
| --- | --- | --- |
| **11** | Healthcare personnel | ❑ |
| **95** | Other (please specify) **ANCHOR** | ❑ |

**17A-18B_2 MOVED EARLIER; 18A/19-21B OMITTED**

22.

How important do you, personally, consider it to be that **all eligible** adult patients receive the **Zoster (shingles) vaccine**?

|  | | |
| --- | --- | --- |
| **1** | 🔾 | Extremely important |
| **2** | 🔾 | Very important |
| **3** | 🔾 | Moderately important |
| **4** | 🔾 | Slightly important |
| **5** | 🔾 | Not at all important |

22a.

How often do you discuss the possible problems from getting sick with shingles if not vaccinated with Zoster, with your **eligible** adult patients?

|  | | |
| --- | --- | --- |
| **1** | 🔾 | Always |
| **2** | 🔾 | Sometimes |
| **3** | 🔾 | Rarely |
| **4** | 🔾 | Never |

23.

To what extent do you agree or disagree with each of the following statements that can be made about the

**Zoster (shingles)** vaccination?

| **Strongly disagree** | | | **Disagree** | **Neutral Agree** | | **Strongly agree** |
| --- | --- | --- | --- | --- | --- | --- |
| **1** | Zoster causes significant burden of disease in my older patients | 🔾 | 🔾 | 🔾 | 🔾 | 🔾 |
| **2** | The burden of Zoster and its complications in older patients is sufficient to make the vaccine important | 🔾 | 🔾 | 🔾 | 🔾 | 🔾 |

**24-27 OMITTED**

# Section D: Tdap Vaccination

Now we would like to focus specifically on the **Tdap (tetanus, diphtheria, pertussis)** vaccine. Please do not include Tetanus and diphtheria (Td).

28.

How knowledgeable would you say you are about the following aspects of the **Tdap (tetanus, diphtheria, pertussis) vaccine**?

| RANDOMIZE **Not at all knowledgeable** | | | **Slightly knowledgeable** | **Moderately knowledgeable** | **Very knowledgeable** | **Fully knowledgeable** |
| --- | --- | --- | --- | --- | --- | --- |
| **1** | Commercial market access coverage | 🔾 | 🔾 | 🔾 | 🔾 | 🔾 |
| **2** | Medicare market access coverage | 🔾 | 🔾 | 🔾 | 🔾 | 🔾 |
| **3** | Which adult patients should receive it | 🔾 | 🔾 | 🔾 | 🔾 | 🔾 |
| **4** | How frequently patients should receive it | 🔾 | 🔾 | 🔾 | 🔾 | 🔾 |

29.

To which of the following groups of **eligible** adult patients do you routinely recommend a **Tdap vaccine**?

|  | | |
| --- | --- | --- |
| **1** | All adults/everybody  **[MUTUALLY EXCLUSIVE]** | 🔾 |
| **98** | None – I don’t recommend this vaccine to adult patients **[MUTUALLY EXCLUSIVE]** | 🔾 |
| **HEADER** | **Depends (select all that apply below)** |  |
| **2** | Patients of a certain age | ❑ |
| **3** | Patients with certain risk factors | ❑ |
| **4** | Patients at risk of getting injured/open wound | ❑ |
| **5** | After an accident/injury | ❑ |
| **95** | Other (please specify) | ❑ |

# ASK IF Q29_2 SELECTED

29a.

You said you recommend a **Tdap vaccine** to patients of a **certain age**. To which of the following age groups do you routinely recommend a Tdap vaccine if no known risk factors? ***Select all that apply***

|  | | |
| --- | --- | --- |
| **1** | Ages 19-29 | ❑ |
| **2** | Ages 30-39 | ❑ |
| **3** | Ages 40-49 | ❑ |
| **4** | Ages 50-59 | ❑ |
| **5** | Ages 60-64 | ❑ |
| **6** | Ages 65 and older | ❑ |

# ASK IF Q29_3 SELECTED

29b.

You said you recommend a **Tdap vaccine** to patients with **certain risk factors**. To which of the following patient types do you recommend a Tdap vaccine? ***Select all that apply***

| **RANDOMIZE** | | |
| --- | --- | --- |
| **1** | Pregnant patients | ❑ |
| **2** | Immuno-compromised conditions | ❑ |
| **3** | Men who have sex with other men | ❑ |
| **4** | Kidney failure / end-stage renal disease / dialysis | ❑ |
| **5** | Heart disease | ❑ |
| **6** | Chronic lung disease (e.g. asthma, COPD) | ❑ |
| **7** | Chronic liver disease (e.g. Hepatitis) | ❑ |
| **8** | Chronic alcoholism | ❑ |
| **9** | Asplenia (non-functioning spleen) | ❑ |
| **10** | Diabetics | ❑ |
| **11** | Healthcare personnel | ❑ |
| **95** | Other (please specify) **ANCHOR** | ❑ |

**32A/33-35B OMITTED**

36.

How important do you, personally, consider it to be that **all eligible** adult patients receive the **Tdap (tetanus, diphtheria, pertussis) vaccine every 10 years**?

|  | | |
| --- | --- | --- |
| **1** | 🔾 | Extremely important |
| **2** | 🔾 | Very important |
| **3** | 🔾 | Moderately important |
| **4** | 🔾 | Slightly important |
| **5** | 🔾 | Not at all important |

36a.

How often do you discuss the possible problems from getting sick with tetanus, diphtheria, pertussis if not vaccinated with Tdap every 10 years, with your **eligible** adult patients?

|  | | |
| --- | --- | --- |
| **1** | 🔾 | Always |
| **2** | 🔾 | Sometimes |
| **3** | 🔾 | Rarely |
| **4** | 🔾 | Never |

37.

To what extent do you agree or disagree with each of the following statements that can be made about the

**Tdap (tetanus, diphtheria, pertussis)** vaccination?

| **Strongly disagree** | | | **Disagree** | **Neutral Agree** | | **Strongly agree** |
| --- | --- | --- | --- | --- | --- | --- |
| **1** | Tetanus, diphtheria and pertussis cause significant burden of disease in my older patients | 🔾 | 🔾 | 🔾 | 🔾 | 🔾 |
| **2** | The burden of tetanus, diphtheria and pertussis is sufficient to make vaccination important | 🔾 | 🔾 | 🔾 | 🔾 | 🔾 |
| **3** | The Tdap vaccine should only be given to patients with certain risk factors | 🔾 | 🔾 | 🔾 | 🔾 | 🔾 |
| **4** | The Tdap vaccine is only needed after a patient has an injury or open wound | 🔾 | 🔾 | 🔾 | 🔾 | 🔾 |

38.

To what extent is each of the following a **barrier** to **eligible** adult patients to receiving the **Tdap (tetanus, diphtheria, pertussis)** vaccination?

| RANDOMIZE  **Barriers to receiving Tdap vaccine Not at all**  **(A)** | | | **Minor (B)** | **Moderate (C)** | **Major (D)** |
| --- | --- | --- | --- | --- | --- |
| **26** | Time window/given every 10 years/patients can’t remember last tetanus shot | 🔾 | 🔾 | 🔾 | 🔾 |
| **28** | Perception it is only needed when patient has an injury or open wound | 🔾 | 🔾 | 🔾 | 🔾 |

**39-41 OMITTED**

**46A/47 OMITTED**

**49-52 OMITTED**

# Section F: Influenza Vaccination

Now we would like to focus specifically on the **Influenza vaccine**.

53.

How knowledgeable would you say you are about the following aspects of the **Influenza vaccine**?

| **RANDOMIZE Not at all knowledgeable** | | | **Slightly knowledgeable** | **Moderately knowledgeable** | **Very knowledgeable** | **Fully knowledgeable** |
| --- | --- | --- | --- | --- | --- | --- |
| **1** | Commercial market access coverage | 🔾 | 🔾 | 🔾 | 🔾 | 🔾 |
| **2** | Medicare market access coverage | 🔾 | 🔾 | 🔾 | 🔾 | 🔾 |
| **3** | Which adult patients should receive it | 🔾 | 🔾 | 🔾 | 🔾 | 🔾 |

54.

To which of the following groups of **eligible** adult patients do you routinely recommend the **Influenza vaccine**?

|  | | |
| --- | --- | --- |
| **1** | All adults/everybody  **[MUTUALLY EXCLUSIVE]** | 🔾 |
| **98** | None – I don’t recommend this vaccine to adult patients **[MUTUALLY EXCLUSIVE]** | 🔾 |
| **HEADER** | **Depends (select all that apply below)** |  |
| **2** | Patients of a certain age | ❑ |
| **3** | Patients with certain risk factors | ❑ |
| **95** | Other (please specify) | ❑ |

# ASK IF Q54_2 SELECTED

54a.

You said you recommend the **Influenza vaccine** to patients of a **certain age**. To which of the following age groups do you routinely recommend the Influenza vaccine if no known risk factors?

## Select all that apply

|  | | |
| --- | --- | --- |
| **1** | Ages 19-29 | ❑ |
| **2** | Ages 30-39 | ❑ |
| **3** | Ages 40-49 | ❑ |
| **4** | Ages 50-59 | ❑ |
| **5** | Ages 60-64 | ❑ |
| **6** | Ages 65 and older | ❑ |

**ASK IF Q54_3 SELECTED**

54b.

You said you recommend the **Influenza vaccine** to patients with **certain risk factors**. To which of the following patient types do you recommend the Influenza vaccine?

## Select all that apply

| **RANDOMIZE** | | |
| --- | --- | --- |
| **1** | Pregnant patients | ❑ |
| **2** | Immuno-compromised conditions | ❑ |
| **3** | Men who have sex with other men | ❑ |
| **4** | Kidney failure / end-stage renal disease / dialysis | ❑ |
| **5** | Heart disease | ❑ |
| **6** | Chronic lung disease (e.g. asthma, COPD) | ❑ |
| **7** | Chronic liver disease (e.g. Hepatitis) | ❑ |
| **8** | Chronic alcoholism | ❑ |
| **9** | Asplenia (non-functioning spleen) | ❑ |
| **10** | Diabetics | ❑ |
| **11** | Healthcare personnel | ❑ |
| **95** | Other (please specify) ANCHOR | ❑ |

**58/59 OMITTED**

60.

How important do you, personally, consider it to be that **all eligible** adult patients receive the **Influenza vaccine (during months surrounding flu season)**?

|  | | |
| --- | --- | --- |
| **1** | 🔾 | Extremely important |
| **2** | 🔾 | Very important |
| **3** | 🔾 | Moderately important |
| **4** | 🔾 | Slightly important |
| **5** | 🔾 | Not at all important |

60a.

How often do you discuss the possible problems from getting sick with the flu **(during months surrounding flu season)** if not vaccinated with Influenza, with your **eligible** adult patients?

|  | | |
| --- | --- | --- |
| **1** | 🔾 | Always |
| **2** | 🔾 | Sometimes |
| **3** | 🔾 | Rarely |
| **4** | 🔾 | Never |

- 1. **OMITTED**

# Section G: Physician & Practice Demographics

Finally we would like some more information about you and your practice G1. Please indicate your gender.

|  | | |
| --- | --- | --- |
| **1** | 🔾 | Male |
| **2** | 🔾 | Female |

G2. Please enter your age. years old **RANGE: 18-87**

- - - Prefer not to answer **[MUTUALLY EXCLUSIVE]**

G2a.

Thinking of all your adult patients **aged 19 years or older**, what proportion has had each of the following diseases in the past year?

## Enter number from 0-100% for each condition; if multiple times in the past year, please count once. Your responses can add to less than or greater than 100.

| **RANGE FOR EACH: 0–100 % of patients 19**  **years or older** | | |
| --- | --- | --- |
| **1** | Shingles | % |
| **2** | Tetanus | % |
| **3** | Diphtheria | % |
| **4** | Pertussis | % |
| **5** | Pneumococcal Pneumonia | % |
| **6** | Influenza | % |

G3.

Thinking of your **Medicare** patients **aged 65+**, approximately what percent of these patients have the following?

# Your total must sum to 100%.

| **RANGE FOR EACH: 0–100 % of patients 65**  **years or older** | | |
| --- | --- | --- |
| **1** | Medicare | % |
| **2** | Medicare and Commercial (supplemental such as AARP) | % |
| **3** | Medicare advantage | % |
|  |  | **TOTAL= 100%** |

G3a.

You may or may not know the **Tdap** vaccine is covered by Medicare Part D. What impact, if any, would having it covered under Part B instead of Part D affect your willingness to administer the vaccine in your practice?

|  | | |
| --- | --- | --- |
| **1** | 🔾 | Much more likely to administer |
| **2** | 🔾 | Somewhat more likely to administer |
| **3** | 🔾 | No impact |
| **98** | 🔾 | Don’t know |

G3b.

You may or may not know the **Zoster** vaccine is covered by Medicare Part D. What impact, if any, would having it covered under Part B instead of Part D affect your willingness to administer the vaccine in your practice?

|  | | |
| --- | --- | --- |
| **1** | 🔾 | Much more likely to administer |
| **2** | 🔾 | Somewhat more likely to administer |
| **3** | 🔾 | No impact |
| **98** | 🔾 | Don’t know |

G4a.

What approach is used at the location where you work to document vaccine administration?

## Select all that apply

|  |  |  |
| --- | --- | --- |
| **1** | ❑ | Electronic Health Records (EHR)/Electronic Medical Records (EMR) |
| **2** | ❑ | Paper Medical Records |
| **3** | ❑ | Immunization Information Systems (IISs) |
| **95** | ❑ | Other (specify) **INSERT TEXT BOX** |

**ASK IF G4A_1**

G4b.

Does your practice use EHR/EMR alerts to assess vaccination status for your adult patients?

|  | | |
| --- | --- | --- |
| **1** | 🔾 | Yes |
| **2** | 🔾 | No |

# ASK IF G4A_1

G4c.

Where in your EHR/EMR system is the information about vaccination status for your adult patients?

|  | | |
| --- | --- | --- |
| **1** | 🔾 | On the first screen, I have to confirm that I have reviewed it before I can proceed |
| **2** | 🔾 | On a later screen, I have to confirm that I have reviewed it before I can exit the patient record |
| **3** | 🔾 | On its own screen, but I do not have to review it |
| **4** | 🔾 | Included in the EHR/EMR, not on its own screen but the information is all together |
| **5** | 🔾 | Included in the EHR/EMR, but not all together, information for each vaccine could be saved in different places |
| **6** | 🔾 | Not included in the EHR/EMR |
| **95** | 🔾 | Other (specify) |

G4d.

Thinking of all of the adult patients, aged 19 years or older, registered at your practice/with you, approximately what proportion of these patients with each type of insurance will you see in a typical year?

## Your responses do not need to equal 100%.

| RANGE: 0-100 FOR EACH  **% patients see in a typical year** | | |
| --- | --- | --- |
| **1** | Medicare patients | % |

| RANGE: 0-100 FOR EACH  **% patients see in a typical year** | | |
| --- | --- | --- |
| **2** | Commercial insurance patients | % |

G5.

Within your practice, how involved are you in each of the following activities surrounding adult immunization policies and practices?

| **RANDOMIZE** | **Not at all involved** | **Slightly involved** | **Moderately involved** | **Very involved** | **Fully involved** |
| --- | --- | --- | --- | --- | --- |
| Setting office-wide immunization practices/protocols | 🔾 | 🔾 | 🔾 | 🔾 | 🔾 |
| Determining office vaccination schedules | 🔾 | 🔾 | 🔾 | 🔾 | 🔾 |
| Selecting vaccines | 🔾 | 🔾 | 🔾 | 🔾 | 🔾 |

G6a.

Which of the following best describes your **practice**?

|  | | |
| --- | --- | --- |
| **1** | 🔾 | Independent, not owned by another healthcare entity |
| **2** | 🔾 | Part of an integrated health system that includes hospitals, practices, and other healthcare settings |
| **3** | 🔾 | Part of an integrated health system that includes other practices like yours only; but not inclusive of hospitals or other healthcare settings (e.g. large medical group) |
| **95** | 🔾 | Other (specify) |

G7.

During the course of this survey, an adverse event (based on FDA guidelines) may have been mentioned which we are required to report to our sponsor’s product safety department.

May we also provide your name and contact information to help them investigate the adverse event more fully?

|  | | |
| --- | --- | --- |
| **1** | 🔾 | Yes |
| **2** | 🔾 | No |

G8.

The researchers who designed this study would like to hear your feedback about this survey, including how easy or difficult it was to take. Please provide any feedback you have below.

# OPEN END

Thank you for completing our survey.
